# Flexible Monolithic 3D-Integrated Self-Powered Tactile Sensing Array Based on Holey MXene Paste

**DOI:** 10.1007/s40820-025-01924-9

**Published:** 2025-09-15

**Authors:** Mengjie Wang, Chen Chen, Yuhang Zhang, Yanan Ma, Li Xu, Dan-Dan Wu, Bowen Gao, Aoyun Song, Li Wen, Yongfa Cheng, Siliang Wang, Yang Yue

**Affiliations:** 1 Information Materials and Intelligent Sensing Laboratory of Anhui Province, Industry-Education-Research Institute of Advanced Materials and Technology for Integrated Circuits, Institutes of Physical Science and Information Technology, Anhui University, Hefei, 230601 People’s Republic of China; 2 Hubei Key Laboratory of Energy Storage and Power Battery, School of Optoelectronic Engineering, Hubei University of Automotive Technology, Shiyan, 442002 People’s Republic of China; 3 Department of Materials Science and Engineering, Northwestern University, 2145 Sheridan Road, Evanston, IL 60208 USA

**Keywords:** Holey MXene, Microsupercapacitor, Tactile sensor, Monolithic 3D integration, Deep learning algorithm

## Abstract

Flexible electronics face critical challenges in achieving monolithic three-dimensional (3D) integration, including material compatibility, structural stability, and scalable fabrication methods. Inspired by the tactile sensing mechanism of the human skin, we have developed a flexible monolithic 3D-integrated tactile sensing system based on a holey MXene paste, where each vertical one-body unit simultaneously functions as a microsupercapacitor and pressure sensor. The in-plane mesopores of MXene significantly improve ion accessibility, mitigate the self-stacking of nanosheets, and allow the holey MXene to multifunctionally act as a sensing material, an active electrode, and a conductive interconnect, thus drastically reducing the interface mismatch and enhancing the mechanical robustness. Furthermore, we fabricate a large-scale device using a blade-coating and stamping method, which demonstrates excellent mechanical flexibility, low-power consumption, rapid response, and stable long-term operation. As a proof-of-concept application, we integrate our sensing array into a smart access control system, leveraging deep learning to accurately identify users based on their unique pressing behaviors. This study provides a promising approach for designing highly integrated, intelligent, and flexible electronic systems for advanced human–computer interactions and personalized electronics.

**Supplementary Information:**

The online version contains supplementary material available at 10.1007/s40820-025-01924-9.

## Highlights

- A flexible monolithic 3D-integrated tactile sensing system, inspired by the tactile perception mechanism of human skin, was developed based on a holey MXene paste.
- Large-scale device fabrication was achieved using blade-coating and imprinting methods, demonstrating excellent mechanical flexibility, low-power consumption, fast response, and stable long-term performance.
- The sensor array was integrated into a smart access control system, leveraging deep learning to achieve precise identification based on the unique pressing behaviors of users.

**Supplementary Information:**

The online version contains supplementary material available at 10.1007/s40820-025-01924-9.

## Graphical abstract

**Figure:**
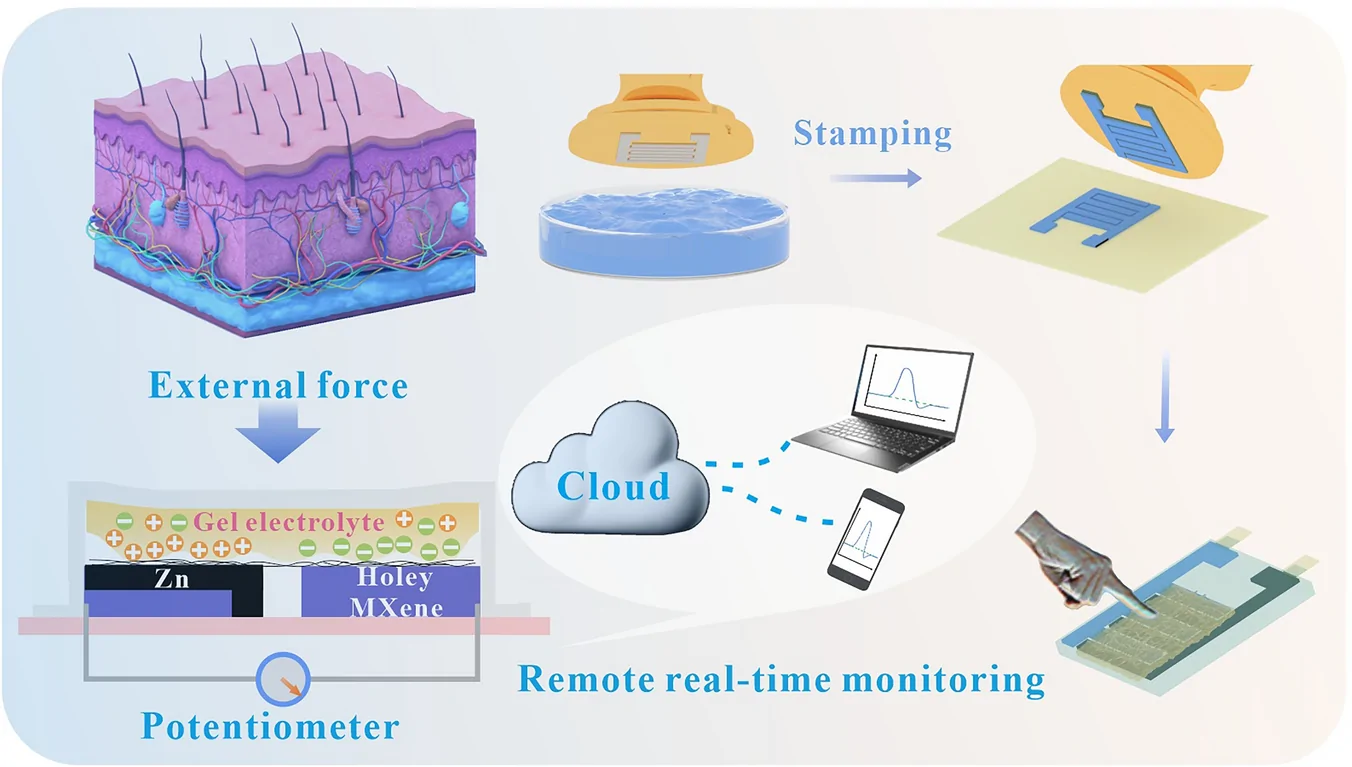

## Introduction

With the trend toward the miniaturization and portability of flexible electronic devices, the integration of energy storage/supply units (e.g., microsupercapacitors and microbatteries) and energy-consuming units (e.g., pressure sensors) into a single 2D plane has gained widespread attention [1–4]. This integration offers numerous advantages such as simplicity, compatibility, and reliability. To accommodate diverse application scenarios such as e-skin, high-precision monitoring, and intelligent transportation, it is essential to further improve spatial resolution, detection accuracy, and system reliability by arraying flexible electronic units [5–10]. Monolithic integrations containing multiple functional units have been reassembled into a 2D planar array, resulting in excessively large array areas, complicated connections, and limited space utilization [11–13]. These issues contradict the fundamental objectives of miniaturization and integration in flexible electronics and hinder the transition from lab-scale research to industrial-scale deployment. Therefore, there is an urgent need to develop new integration strategies for practical application.

The nanofabrication of integrated circuits has gradually approached its physical limits. To expand Moore’s law, 3D integration technologies that vertically stack multiple transistors can allow more functional units to be well-assembled in a smaller footprint, realizing a higher level of integration while preserving their excellent performance [14, 15]. By applying this concept to flexible electronic devices, monolithic 3D-integrated flexible electronic devices can be constructed through vertical integration of functional units combined with a planar array design. This approach not only significantly reduces the effective space but also reduces signal transmission delays. In contrast to conventional rigid electronics, flexible integrated units must maintain structural integrity and functionality when subjected to mechanical deformation [16–19]. However, the poor compatibility between different materials and functional units directly leads to weak interconnections, mechanical mismatches, and structural instability. In particular, when different functional units are vertically integrated, the excessive interface layers caused by the complex unit structures make it difficult to maintain good adhesion and structural integrity under external mechanical stimulation. Owing to the functional couplings of the layer–layer interfaces, it is challenging to match the microstructure, moduli, and properties of different material structures [20–22]. In addition, traditional micro/nanomanufacturing assembly techniques have relatively cumbersome drawbacks owing to the lack of effective and continuous connections with external circuits when simultaneously constructing different components, making it challenging to ensure optimal system compatibility [23, 24]. Thus, the construction of multifunctional and highly monolithic 3D-integrated electronic devices is challenging in terms of material selection, device structure design, process engineering, and circuit connections [25]. Addressing these challenges requires the cooperation and development of many disciplines, such as physics, materials science, and electronics [26, 27].

Ti_3_C_2_T_x_ MXenes exhibit exceptional intrinsic conductivity, richly tunable surface chemistry, superior mechanical flexibility, and unique dual-functionality for energy storage and sensing [28, 29]. These properties collectively satisfy the stringent requirements for active materials in high-performance flexible monolithic 3D-integrated tactile sensing arrays [30–33]. Compared to pristine MXene, the in-planar mesoporous structure of holey MXene overcomes the limited active sites and high tortuosity of the ionic transport paths caused by the self-stacking of nanosheets. Above all, holey MXene with multiple functions can simultaneously serve as a sensing material, an active electrode, a collector, and a conductive interconnect, effectively enhancing the matching degree of different functional units when vertically integrated into an all-in-one system. Thus, holey MXene is extremely suitable as an active material for constructing monolithic 3D-integrated sensing systems with high matching, integration, and flexibility.

The tactile perception of human skin primarily relies on various types of receptors, such as Merkel cells, distributed in the dermis layer, which play a key role in activating the response behavior of mechanically gated ion channels. Drawing on the tactile mechanism of human skin, holey MXene was selected to construct a vertical one-body unit (VOU) that can be simultaneously used as a microsupercapacitor and pressure sensor. The monolithic 3D integration of VOUs based on the same active material significantly reduces the number of vertically oriented interfaces, resulting in a higher density of functional units in the same footprint. When combined with highly consistent materials, it shortens the interconnect paths, reduces power consumption, and improves integration and matching (Fig. S1). Specifically, we developed a holey MXene paste regulated by an interlayer force regulation mechanism to scrape or stamp it onto an arbitrary substrate for monolithic 3D integration. As a proof-of-concept, by combining a monolithic 3D-integrated sensing system with deep learning algorithms, a smart access control user identification system was constructed to precisely recognize and verify user identity. The monolithic, 3D vertically integrated, all-in-one strategy proposed in this study provides a new paradigm for the highly integrated and intelligent development of flexible electronic systems.

## Experimental Section

### Materials

High-purity Ti_3_AlC_2_ particles were purchased from Laizhou Kai Kai Ceramic Materials Co., Ltd. 30% H_2_O_2_ (A.R. grade), LiF (A.R. grade), KCl (A.R. grade), HCl (A.R. grade), and ZnSO_4_·7H_2_O (A.R. grade) were obtained from Sinopharm Group Co., Ltd. Cellulose nanofiber (CNF) solutions were purchased from Guilin Qihong Technology, and gelatin was purchased from Ourchem Ltd. All chemical reagents were used as received without further purification.

### Preparation of Holey MXene Paste

The detailed preparation process of holey MXene nanosheets is described in our previous work [34–36]. The final holey MXene nanosheets were evenly dispersed in a solution. Flocculation was observed when HCl was added to the solution. The holey MXene paste obtained from the above mixture was centrifuged at a high speed. The degree of slight flocculation was regulated by the HCl content. More HCl led to the severe flocculation of the holey MXene paste. Subsequently, we designed and fabricated various holey MXene-based patterns by scraping and stamping the paste onto paper, followed by drying at room temperature (25 °C).

### Preparation of Gel Electrolyte

Briefly, 3 g of gelatin was slowly added into 30 mL of a ZnSO_4_ solution (1 M) and stirred thoroughly at 75 °C. Once the mixture was fully dissolved, it was injected into a suitable container and cooled to room temperature (25 °C) to obtain a gel electrolyte. The thickness of the gel was adjusted by adjusting the injection volume.

### Assembly of the VOU

First, a holey MXene-based paper was engraved into interdigital electrodes using a laser engraving technique; then, zinc was electrodeposited on the surface of the holey MXene as an active electrode [37]. Subsequently, 100 μL of a CNF solution (5 mg mL^−1^) was sprayed on the surface of the interdigital electrodes as a barrier layer. Then, a gel electrolyte was carefully placed on the surface of the CNF fibers, and a copper tape was connected to both sides of the electrode to establish an external circuit. Finally, the VOU was assembled using polyethylene terephthalate (PET) as the encapsulation layer.

### Characterization

The holey MXene nanosheets were observed using a double spherical aberration electron microscope (FEI Titan Themis Z). The crystal structures of the samples were determined using X-ray diffraction (XRD; SmartLab). The chemical bond valences and compositions of the samples were determined using X-ray photoelectron spectroscopy (XPS; ESCALAB 250Xi). Raman spectra were obtained using inVia-Reflex equipment. Fourier-transform infrared (FTIR) spectroscopy was performed using a Vertex 80 + Hyperion 2000 instrument.

### Performance Testing

The test system consisted of a motion control system (7SC306), single-axis displacement Table (7STA02100B), dynamometer (HANDPI), and multimeter (Keithley DMM7510). The entire system was connected to a computer and controlled by it. Cyclic voltammetry (CV), galvanostatic charge/discharge (GCD), and electrochemical impedance spectroscopy (EIS) curves of the devices were obtained using an electrochemical workstation (CHI 760E). Their anti-self-discharge performance was tested using open-circuit voltage (OCV) and GCD tests.

### Finite Element Analysis (FEA)

The radial compression behavior and internal stress distribution of the VOU were obtained using COMSOL software. A 2D planar model was used to simulate the barrier layer, which consisted of a hemispherical gel structure in the upper layer and a fibrous structure in the lower layer to form a continuum model. A solid-mechanics solution was obtained under reasonable boundary conditions and mesh delineation. The lower boundary of the model was used as a fixed boundary, and a vertical uniform stress was applied to the upper boundary, from which the internal stress distribution was obtained. The solid-mechanics problem was solved under appropriate boundary conditions and mesh divisions. Specifically, the lower bound was fixed, and a uniform vertical stress was applied to the upper bound. The internal stress distribution was obtained after solving the equations under the aforementioned conditions.

### Design of the Access Control User Identification System

The access control user identification system consists of a wireless transmission module, host computer application, and a monolithic 3D-integrated sensing system. A host computer application was developed using the LabVIEW software. The measured data from the monolithic 3D-integrated sensing system were transmitted to a computer via a wireless transmission module. When a user enters the password, dynamic pressing information is collected in real time using the host application. The features were extracted using MATLAB and fed into a backpropagation (BP) neural network model for recognition. The user’s name was displayed according to the correct feature feedback. For the deep learning algorithm, the accuracy of the neural network was set to 2.5, and the number of hidden layers was set to 40 nodes; furthermore, the number of iterations was set to 5000. These parameters can be adjusted to achieve higher accuracy while simultaneously preventing the network from overfitting. Instead of the traditional gradient descent algorithm, the Adam optimization algorithm was used for training data to achieve faster and easier convergence.

## Results and Discussion

### Monolithic 3D Integration of VOU toward the Flexible Tactile Sensing System

**Fig. 1:**
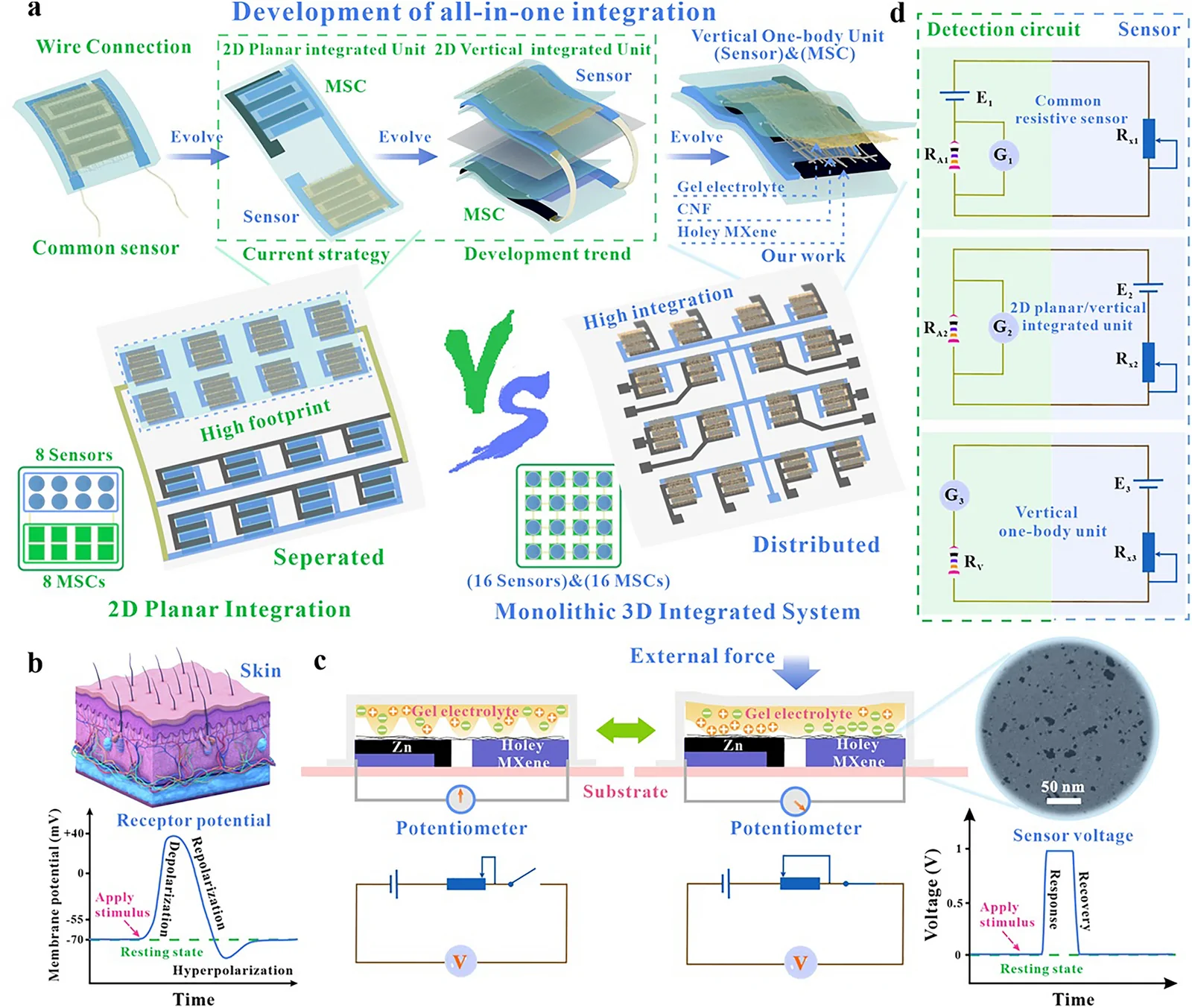
Monolithic 3D integration of VOU toward the flexible tactile sensing system. **a** Development of the all-in-one integration and the advantages of 3D vertically integrated systems. **b** Working process and principle of the skin responsive to the external stimuli. **c** Ion distributions and circuit diagram of the VOU responsive to force. **d** Comparisons of the equivalent circuit diagrams of common resistive sensors, 2D planar/vertical integrated units, and VOUs when detecting the external pressure, where the detection circuit is colored in yellow and the sensor in blue

In Fig. 1a, the self-powered unit consists mainly of a microsupercapacitor (MSC) and a sensor. The common strategy was derived from direct wire connection of the two devices to planar integration on a single substrate. The next step in development is to construct 2D vertically integrated units to further enhance integration. The VOU proposed in this study can realize the functions of the two devices simultaneously, thereby significantly reducing the number of interfaces in the vertical direction compared with the direct 2D vertical integration of the two devices. In particular, 2D planar integration can only be achieved by constructing the MSCs and sensors separately when building arrays. For 3D vertical designs, it is easy to obtain arrays of units that simultaneously function as MSCs and sensors. Consequently, more devices can be integrated into the same footprint, with simpler wiring and lower power consumption.

The mechanism of the VOU is inspired by the tactile sensing system of the skin (Fig. 1b). When an external stimulus is applied to the skin, mechanically gated ion channels in the sensory cells open. This allows the ions to migrate or flow across the cell membrane, thereby creating a physiological electrical signal (membrane potential). When a mechanical stimulus is released, the membrane potential returns to its initial level by pumping specific ions back into the cell membrane. This efficient and low-energy sensing system for the human skin provides a new strategy for the design of flexible sensing units. In this study, a flexible VOU consisting mainly of two different electrodes (holey MXene and electroplated zinc nanoparticles) and a gel electrolyte with a surface microstructure was constructed (Fig. S2). The holey MXene maintained the same excellent hydrophilicity as the pure MXene (Fig. S3). The Tyndall effect indicated the good dispersion of both the MXene and holey MXene solutions (Fig. S4). HAADF-STEM images clearly revealed the presence of abundant in-plane mesopores on the nanosheets (Fig. S5a, b). The XRD patterns showed that the (002) peak position of holey MXene remained nearly unchanged compared to that of MXene; the peak intensity decreased slightly, but the peak width increased slightly, suggesting that the introduction of in-plane defects reduced the stacking order of the nanosheets to some extent (Fig. S6). In the C 1*s* XPS spectrum, the C–C peak of the holey MXene was significantly stronger, indicating that the introduction of mesopores exposes more carbon atoms on the surface. The C-Ti peak remained intact, confirming the preservation of the layered structure (Fig. S7). The Ti 2*p* XPS spectrum of the holey MXene showed a weak Ti–O peak, indicating the effective removal of TiO_2_ (Fig. S7). In the FTIR spectrum, the holey MXene exhibited three distinct characteristic peaks corresponding to -OH, -C = O, and -Ti–O (Fig. S8). The Raman spectra revealed more prominent D and G bands in the holey MXene, which were attributed to carbon formation during H_2_O_2_ etching (Fig. S9). These characterization results confirmed the successful synthesis and structural features of the holey MXene material (Figs. S3–S9). When a VOU was mechanically stimulated by an external force, the contact area between the gel electrolyte and electrode increased, leading to the formation of more ion channels and enhanced ion migration. As a result, charge migration occurred in the external circuit to maintain the charge balance. Thus, the pressure signal was successfully converted and encoded into a change in the electrical signal of the external circuit. When the mechanical stimulation was removed, the ion channel returned to its initial state under the action of the electrode/electrolyte interface. Consequently, the ion migration diminished, and the electrical signal in the external circuit weakened correspondingly (Fig. 1c). The VOU significantly reduces the number of vertical layer interfaces between materials and structures, providing high efficiency, low-energy consumption, and excellent sensing capabilities. In addition, the holey MXene provides abundant active sites and a short ion transport distance through the in-plane mesopores, effectively improving the ion mobility and specific capacity of the active electrode. Holey MXenes with multiple functions can be simultaneously used as flexible electrodes, sensing materials, collectors, and conductive interconnects, preventing the interfacial coupling of different materials. Thus, it effectively improves the degree of matching of different functional units when vertically integrated into 3D-integrated systems.

To further understand the operating principle and power consumption of the VOU, equivalent circuit diagrams of a common piezoresistive sensor, a 2D planar/vertical integrated unit, and the VOU are discussed and analyzed for detecting pressure signals (Fig. 1d). Piezoresistive sensors require an external power supply (*E*_*1*_) to detect the pressure signals, resulting in relatively high-power consumption due to the internal resistance of the ammeter (*R*_*A1*_) and low internal resistance of the sensitive material (*R*_*X1*_), which are unfavorable for the practical application of flexible microelectronics. Although development of the 2D planar/vertical integrated unit eliminates the need for an external power supply, the circuit still contains a low-resistance ammeter (*R*_*A2*_) and highly conductive sensitive element (*R*_*X2*_). The low resistance of the overall circuit results in high-power consumption. For the VOU, the detected voltage (*V*) varied with the resistance of the sensing unit, as shown in the following equation:

$$V={E}_{3}\frac{{R}_{V}}{{R}_{V}+{R}_{X3}}$$

Here, *E*_*3*_ is the potential difference between the two electrodes of the device, *R*_*V*_ is the internal resistance of the voltmeter (10 MΩ), *R*_*X3*_ is the internal resistance of the device, and *V* is the test voltage of the VOU. The internal resistance *R*_*X3*_ is relatively large without loading pressure and is close to infinity; therefore, the overall power consumption of the device is close to 0, and the device is in a silent state. As the external pressure increases, the number of migrated ions in the device increases, and *R*_*X3*_ decreases; furthermore, the output voltage gradually approaches *E*_*3*_. At this point, the instantaneous power consumption theoretically reaches its peak value. However, because the total resistance in the entire circuit is mainly determined by *R*_*V*_, the generated current is extremely small. Thus, the overall power consumption remains at an extremely low level, and the device maintains its excellent low-power performance even under more intense mechanical stimulation. Thus, the VOU has the unique advantages of low-power consumption and high integration, which have promoted the development of flexible all-in-one integration electronics.

### Overall Study on the Holey MXene Paste

**Fig. 2:**
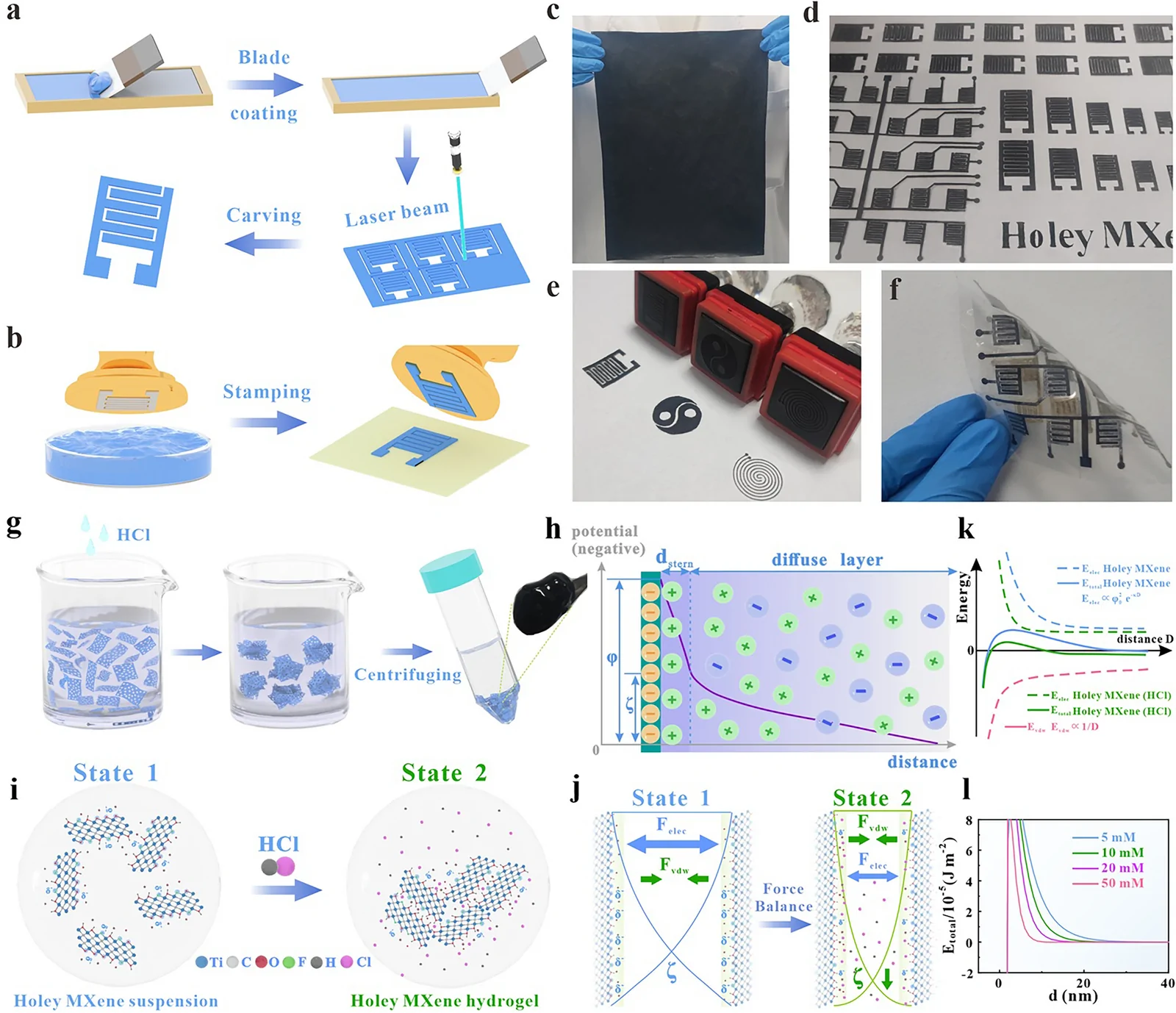
Overall study on the holey MXene paste. **a, b** Schematic depicts the fabrication process of holey MXene-based interdigital electrodes. **c, d** Holey MXene-based A4 paper is fabricated and then engraved into various electrodes and arrays as required. **e** Digital images of the holey MXene-based patterns fabricated by the stamping method. **f** Holey MXene-based paper array exhibits excellent flexibility. **g** Forming process of the holey MXene paste. **h** Electric double-layer model of the holey MXene and the corresponding potential distribution after adding HCl. **i** Potential energy change of the holey MXene before and after adding HCl. **j, k** Change of interlayer spacing between the holey MXene nanosheets under the action of the microscopic force (*E*_*elec*_ represents the electrostatic repulsive potential energy, *E*_*vdw*_ represents the van der Waals attractive potential energy, and *E*_*total*_ represents the total potential energy). **l** Changes of total potential energy and layer spacing with HCl concentration from 5 to 50 mM

The rapid development of flexible sensing integration has put forward higher demands on fabrication and assembly efficiency. In this study, a holey MXene paste was prepared by utilizing the slight flocculation phenomenon regulated by the interlayer force regulation mechanism, which can be extended to various manufacturing techniques. Thus, flexible MXene-based units and arrays were constructed by paste blade scraping and stamping onto a substrate (Fig. 2a, b; see the Experimental section for the detailed preparation processes). To explore the feasibility of up-scaling, the holey MXene paste was uniformly scraped onto a full A4 sheet by blade scraping; the entire sheet showed good electrical conductivity (Figs. 2c and S10). Multiple patterns and arrays of specific sizes could be manufactured by laser engraving the MXene-based paper, as shown in Fig. 2d. Additionally, electrodes could be quickly stamped into customized shapes, such as fork-finger, tai chi, and spiral shapes (Fig. 2e). Moreover, 4 × 4 and 3 × 3 arrays based on the holey MXene paper were fabricated and assembled into monolithic 3D-integrated sensing systems within a short duration (Figs. 2f and S11).

To prepare the holey MXene paste accurately, the regulation mechanism of slight flocculation was further investigated. This flocculation of the holey MXene originated from the addition of H^+^, and a paste was obtained after multiple centrifugations (Fig. 2g). The regulation mechanism of the interlayer force can be explained by the electric double-layer (EDL) theory, which accounts for the observed slight flocculation. An EDL is characterized by the finite thickness of the Stern and diffuse layers in a Gay–Chapman–Stern (GCS) model, as shown in Fig. 2h [38–40]. The thickness of the EDL represents the range of influence of the surface charge. The interlayer distance of the nanosheets depends on the competition between the electrostatic repulsion potential energy and van der Waals potential energy. Holey MXene nanosheets have a negative charge in deionized water owing to the ionization of surface -OH groups (zeta potential of − 51 mV), resulting in the strong electrostatic repulsion between adjacent nanosheets and a uniformly dispersed solution (Fig. 2i, j, state 1). Upon introducing small amounts of H⁺, the zeta potential gradually reaches −28 mV (Fig. S12). This is attributed to the inhibition of -OH ionization on the MXene surface by H^+^, which reduces the surface charge density. This electrostatic shielding effect compresses the thickness of the EDL, significantly weakening long-range electrostatic repulsion. The van der Waals force becomes dominant, resulting in a significant reduction in the interlayer spacing between the nanosheets (Fig. 2i, j, State 2). When the electrostatic repulsion and van der Waals forces reach a balance, a slightly flocculated holey MXene paste is formed (Fig. 2k). Additionally, we calculated the total potential energy at different H^+^ concentrations, and the zero point of the total potential energy corresponded to the balanced state of the two forces (Fig. 2l). These results indicate that an increase in the H^+^ concentration reduces the distance D corresponding to the balance point, thereby exacerbating the degree of flocculation. Therefore, we strictly controlled the H^+^ concentration to avoid disrupting the stability of the paste.

### Cyclic Stability and Overall Component Degradability of the VOU

**Fig. 3:**
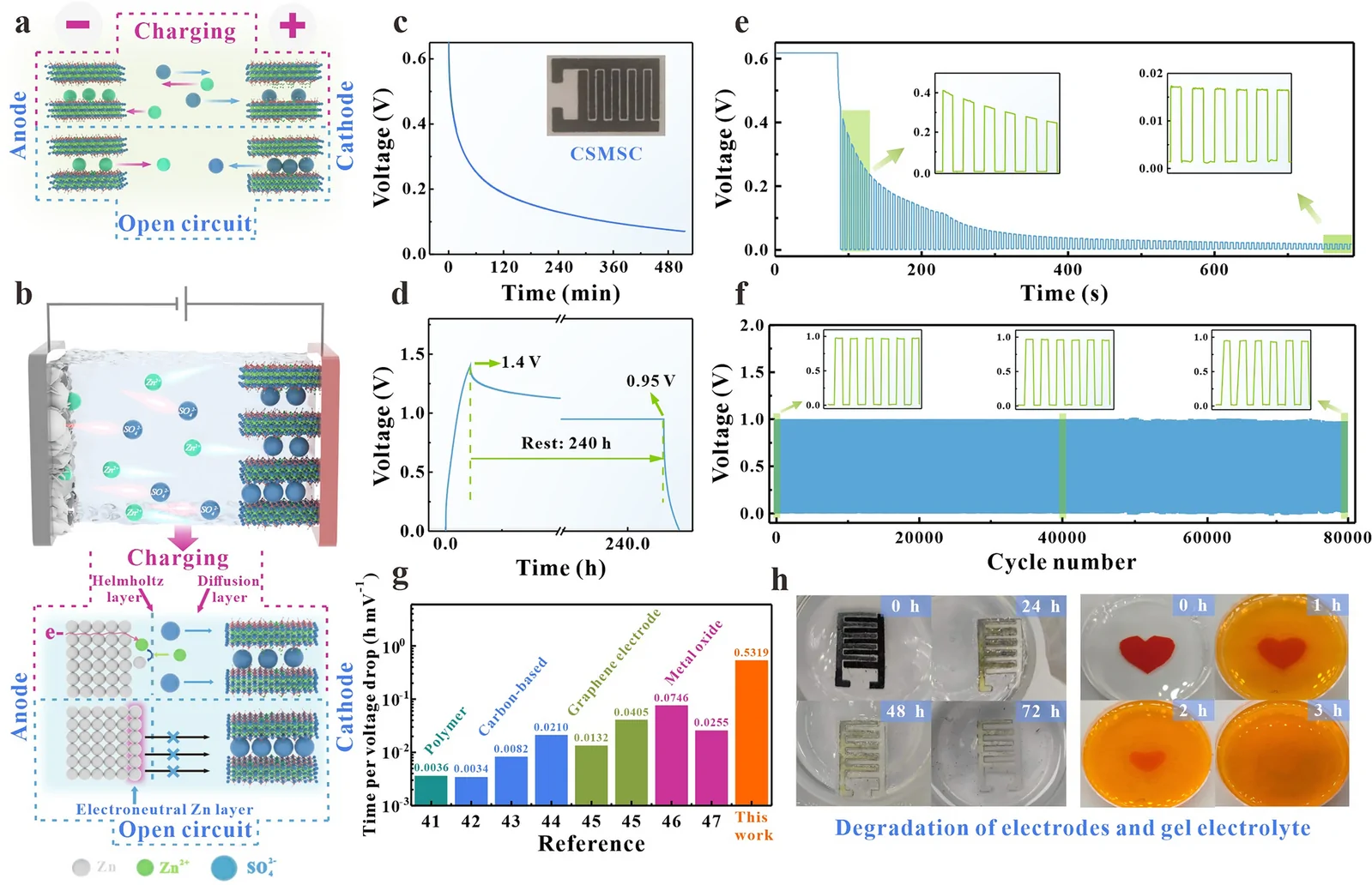
Cyclic stability and overall component degradability of the VOU. **a, b** Comparisons of the discharging principle between the CSMSC and the ZIMSC. **c, d** Comparisons of the self-discharge performance of the VOU-CSMSC and the VOU-ZIMSC. The cycling stability of **e** VOU-CSMSC and **f** VOU-ZIMSC after charging. **g** Comparisons of the anti-self-discharge rates of our work with the previously reported devices. **h** Whole component degradability of the VOU

MSCs are commonly used to power flexible pressure sensors because of their facile integration and excellent electrochemical performance. However, the severe self-discharge behavior of conventional symmetric microsupercapacitors (CSMSCs) can lead to an unstable voltage, hindering the long-term operation of wearable devices (Fig. 3a). In zinc-ion microsupercapacitors (ZIMSCs), the valence state transition of Zn^2+^ plays a key role in resistance to self-discharge. Upon charging, Zn^2+^ ions migrate toward the anode, where they are reduced to Zn atoms and then immobilized on the Zn electrode. Once in an open-circuit state, the electrically neutral Zn atoms are no longer able to migrate, thus suppressing the self-discharge behavior of the ZIMSCs (Fig. 3b). To confirm this mechanism, we assembled VOUs based on the CSMSC and ZIMSC (VOU-CSMSC and VOU-ZIMSC, respectively) for self-discharge testing. The VOU-CSMSCs exhibited obvious self-discharge behavior, whereas the VOU-ZIMSCs maintained good electrochemical performance after 240 h (Figs. 3c, d, and S13). The OCV only dropped from 1.40 to 0.95 V with a low self-discharge rate of 1.875 mV h^−1^. Compared with previously reported works, the holey MXene-based VOU-ZIMSCs possess excellent anti-self-discharge performance (Fig. 3g) [41–47]. In addition, the cycling stabilities of the VOU-CSMSCs and VOU-ZIMSCs were also tested after charging. The responding voltage of the VOU-CSMSC decayed rapidly, in agreement with the self-discharge curve shown in Fig. 3e. In contrast, the VOU-ZIMSC exhibited long-term stability over 80,000 cycles with no significant performance degradation (Fig. 3f). Additionally, after every 200 cycles in five sets, the VOU-ZIMSC was subjected to charge–discharge testing, which indicated a capacitance retention rate of 92% (Fig. S14). The use of CNF separators and PET encapsulation enhanced robustness by protecting the active layer from delamination and cracking. Furthermore, SEM images showed that the holey MXene-based VOU maintained the integrity of its surface microstructure under stress and bending conditions (Fig. S15). Considering that environmental protection is becoming increasingly important for the development of human society, the degradation mechanism of the holey MXene-based VOUs was analyzed. The results showed that the holey MXene solution completely degraded after 18 days, and the holey MXene-based paper completely degraded within 72 h of the addition of 5% H_2_O_2_. A residual yellow powder, consisting of TiO_2_ nanoparticles, was obtained. The gel layer rapidly disappeared within 3 h owing to its good water solubility (Fig. 3h, S16, and S17). This indicates that VOUs can be completely degraded, and that they have good biocompatibility with the environment.

### Sensing Performance of the VOUs

**Fig. 4:**
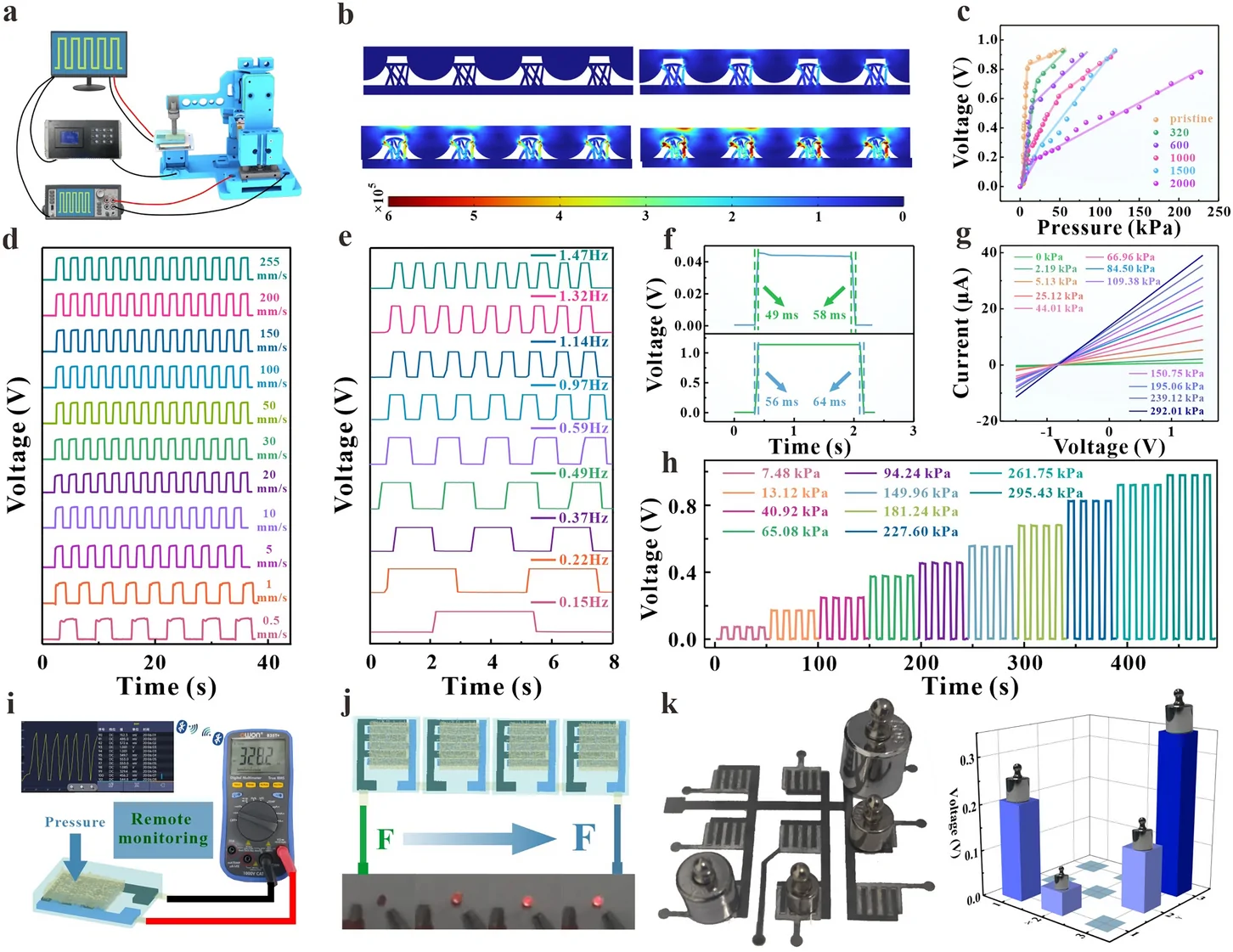
Sensing performance of the VOUs. **a** Schematic diagram of the highly precise electrical test system. **b** Stress distribution simulation of VOU obtained by COMSOL. **c** Sensitivity curves of the VOUs with different roughnesses of the gel electrolyte. The force-electrical response of the VOU under **d** different speeds and **e** different frequencies at 302.31 kPa. **f** Response/recovery time of the VOU at low and high pressures. **g, h** *I-V* and *V-T* curves of the VOU under a series of pressures. **i** Human–computer interactive application of the VOU.** j** Brightness of the LEDs is controlled by the VOU. **k** 3 × 3 monolithic 3D-integrated tactile sensing system for detecting constant pressure distribution

Figure 4a shows a sensing performance test system for the VOUs. The test system consisted of a force sensor, stepping motor, transition stage, and voltmeter. To further improve the sensitivity of the VOU, a gel electrolyte with a surface microstructure fabricated by modeling sandpaper was used to lower the initial voltage. The introduction of in-plane mesopores in MXene effectively increased the number of active sites, enhanced ion accessibility, and improved the electrochemical and sensing performance. This holey design was suitable for constructing vertically stacked monolithic 3D integrations, where each VOU simultaneously functioned as a microsupercapacitor and pressure sensor. This ingenious structural design reduced interface mismatch, shortened interconnection paths, minimized energy loss, and enhanced mechanical strength. On adjusting the contact pressure of the VOU (from 20 to 200 g), the low-frequency region of the Nyquist plot shifted to the left, and the *R*_*Ω*_ value correspondingly decreased, indicating that the impedance of the device decreases significantly with increasing pressure (Fig. S18). COMSOL stress distribution simulations confirmed that as the external pressure increased, the device compressed gradually, and the contact area between the electrolyte and holey MXene-based electrode continued to increase. The vertically stacked structure effectively converted mechanical stimulation into enhanced electrode/electrolyte interactions, resulting in an increase in the measured voltage (Fig. 4b) [48, 49]. In addition, introducing gel electrolytes of different roughness values by modeling sandpaper with different meshes can regulate the switching ratio, linearity, and detection range of the VOU, as shown in Figs. 4c and S19. As the number of sandpaper meshes increased, more complex microstructures were introduced into the gel, which significantly delayed the saturation of the contact area and increased the pressure required to reach saturation. Consequently, both the pressure detection and linear response range of the sensor simultaneously improved with increasing sandpaper mesh number. The VOU fabricated by modeling sandpaper with a 2,000 mesh was selected to study the other sensing performances. The *V-T* curves of the device were tested at different speeds and frequencies at 302.31 kPa, and both showed rapid responses and stability (Fig. 4d, e). The response and recovery times of the VOUs were also measured. Under low pressure, the response and recovery times were 49 and 58 ms, respectively, which were almost the same as those under high pressures (56 and 64 ms, respectively) (Fig. 4f). A response time of less than 100 ms is far below that of various reported pressure sensors (Table S1). The linear *I-V* curves of the VOU were tested in the voltage range of − 1.5 to 1.5 V, and the results indicated good ohmic contact between the electrodes of the gel (Fig. 4g). A series of *V-T* curves of the VOU were systematically measured, as shown in Figs. 4h and S20, demonstrating that the device can effectively distinguish different pressures.

The excellent properties of the VOU can be attributed to its high material matching, microstructure design, and efficient sensing mechanisms. To further explore the practical application of the VOU, it was used in series/parallel connections, human–computer interaction, human signal detection, and other fields, demonstrating good and reliable response signals (Figs. S21–S26). By joining the VOU and Bluetooth multimeters, the received pressure signal was transmitted synchronously to a mobile phone or computer, thus achieving remote real-time transmission in a variety of physical scenarios (Fig. 4i). The VOUs were connected in series to form a complete circuit with an LED lamp. As the pressure gradually increased, the LED lamp became brighter without the need for an external power supply unit. The brightness could be precisely modulated by applying pressure, showing great potential for highly integrated electronic devices (Fig. 4j, Video S1). Finally, the holey MXene paste was used to prepare a flexible monolithic 3D-integrated sensing system based on 3 × 3 arrays, as shown in Fig. 4k. This accurately distinguished the pressure distributions at different locations.

### Application of the Access Control User Identification for the Monolithic 3D-Integrated Tactile Sensing System

**Fig. 5:**
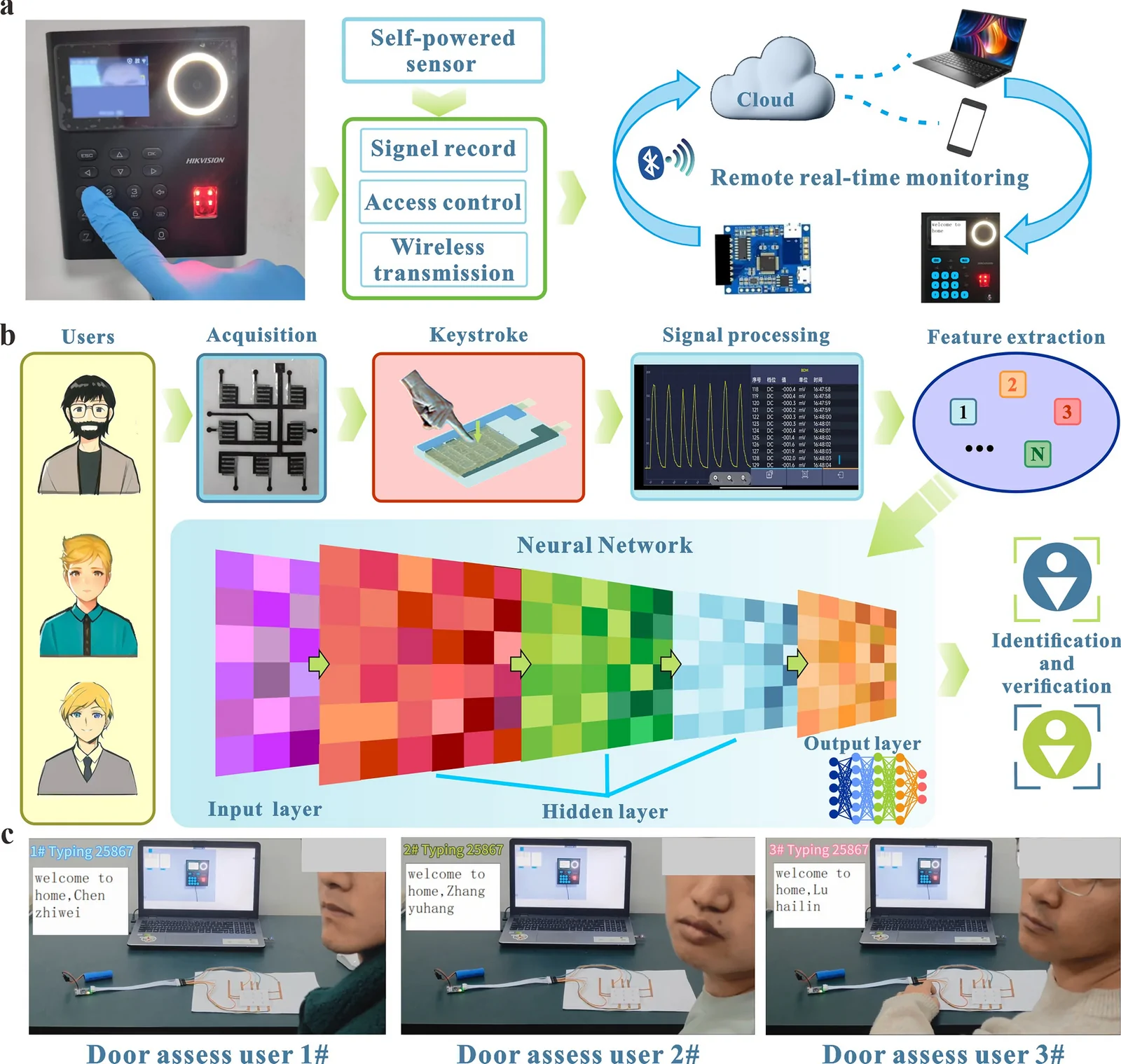
Application of the access control user identification for the monolithic 3D-integrated tactile sensing system. **a** Operational process of the access control user identification system. **b** Flowchart for user feature recognition and verification through the deep learning algorithm. **c** Three users enter the same password, “25,867,” through the sensing system, and the users’ characteristics can be accurately recognized

With the widespread application of human–computer interactions, user identification systems have put forward higher requirements for internet security and privacy security. Typically, the fingerprint peaks and pressing behaviors of everyone are completely different, and this can be used to identify biometric characteristics. A flexible wireless intelligent access control user identification system assembled using the monolithic 3D-integrated sensing system was used to verify a user's identity, as shown in Fig. 5a. The collected data were transmitted by the system and uploaded to the cloud, and the user was identified through deep learning, thus improving the level of security and intelligence (Fig. 5b). The key information of different users was obtained by touching the monolithic 3D-integrated sensing system, and the output electrical signals were collected continuously and simultaneously. The pressing amplitudes varied slightly among the different users, and the durations of holding and touching differed significantly. Personal dynamic behavior characteristics were extracted from digital signals through logical operations, such as peak extraction and filtering. Finally, the extracted features were transmitted to a BP neural network for classification [50, 51]. In order to build the training data sets for the BP neural network model, a digital sequence cipher "25,867" was selected as an example to collect the dynamic behavior features by repeatedly pressing the system. Fourteen features were extracted, including five holding times (high electrical levels), four intervals (low electrical levels), and five signal amplitudes. Among 500 random samples per user, 400 were used as the training set, and the remaining 100 were used as the validation set for user identification. The dynamic characteristics of these typing actions were input into the artificial neural network model to identify a user's specific behavioral information. Successful verification occurred when the same password was entered through the access control system, prompting the display of the message, "Welcome to home, XXX!" (Fig. 5c, Video S2). To better verify the robustness and accuracy of the BP neural network model, a confusion matrix for the user access control system was obtained with an accuracy rate of 98.67%, as shown in Fig. S27. In summary, a monolithic 3D-integrated sensing system that combines an artificial neural network algorithm and access control user identification system can effectively learn, adapt, and recognize the behavioral characteristics of users.

## Conclusion

We successfully demonstrated a flexible monolithic 3D-integrated tactile sensing system based on a multifunctional holey MXene material. The design of the VOU, benefiting from a high degree of material consistency, effectively reduced the number of vertical interfaces, resulting in shorter interconnect paths, lower power consumption, and higher integration and matching. To extend the scalability for industrial production, we developed a holey MXene paste for mass fabrication based on the slight flocculation behavior regulated by the interlayer force regulation mechanism. The integrated tactile sensing system prepared using an efficient, highly matched, and scalable strategy can be applied in various fields of augmented and virtual reality interactions, real-time health monitoring, and personalized medicine, providing a new paradigm for the intelligent development of flexible electronic systems. The monolithic 3D-integrated architecture based on the holey MXene modular design facilitates the integration of other sensors, such as temperature and humidity, in the same vertical stack to build more functional systems.

## Supplementary Information

Below is the link to the electronic supplementary material.

Supplementary file1 (MP4 411 KB)

Supplementary file2 (MP4 4424 KB)

Supplementary file3 (DOCX 12924 KB)

## References

[1] S. Zheng, X. Shi, P. Das, Z.-S. Wu, X. Bao, The road towards planar microbatteries and micro-supercapacitors: from 2D to 3D device geometries. Adv. Mater. **31**(50), 1900583 (2019). 10.1002/adma.201900583 10.1002/adma.201900583 31222810

[2] G. Li, Z. Ma, C. You, G. Huang, E. Song et al., Silicon nanomembrane phototransistor flipped with multifunctional sensors toward smart digital dust. Sci. Adv. **6**(18), eaaz6511 (2020). 10.1126/sciadv.aaz6511 32494679 10.1126/sciadv.aaz6511 PMC7195183

[3] C. Ye, M. Wang, J. Min, R.Y. Tay, H. Lukas et al., A wearable aptamer nanobiosensor for non-invasive female hormone monitoring. Nat. Nanotechnol. **19**(3), 330–337 (2024). 10.1038/s41565-023-01513-0 37770648 10.1038/s41565-023-01513-0 PMC10954395

[4] P. Wang, G. Sun, S. Hua, W. Yu, C. Meng et al., Multifunctional all-nanofiber cloth integrating personal health monitoring and thermal regulation capabilities. InfoMat **7**(1), e12629 (2025). 10.1002/inf2.12629

[5] Y. Li, Z. Tian, X.-Z. Gao, H.-Y. Zhao, X. Li et al., All-weather self-powered intelligent traffic monitoring system based on a conjunction of self-healable piezoresistive sensors and triboelectric nanogenerators. Adv. Funct. Mater. **33**(52), 2308845 (2023). 10.1002/adfm.202308845

[6] J.-Y. Yoo, S. Oh, W. Shalish, W.-Y. Maeng, E. Cerier et al., Wireless broadband acousto-mechanical sensing system for continuous physiological monitoring. Nat. Med. **29**(12), 3137–3148 (2023). 10.1038/s41591-023-02637-5 37973946 10.1038/s41591-023-02637-5

[7] H. Cho, I. Lee, J. Jang, J.-H. Kim, H. Lee et al., Real-time finger motion recognition using skin-conformable electronics. Nat. Electron. **6**(8), 619–629 (2023). 10.1038/s41928-023-01012-z

[8] P. Wang, G. Wang, G. Sun, C. Bao, Y. Li et al., A flexible-integrated multimodal hydrogel-based sensing patch. Nano-Micro Lett. **17**(1), 156 (2025). 10.1007/s40820-025-01656-w 10.1007/s40820-025-01656-w PMC11845634 39982550

[9] P. Wang, X. Li, G. Sun, G. Wang, Q. Han et al., Natural human skin-inspired wearable and breathable nanofiber-based sensors with excellent thermal management functionality. Adv. Fiber Mater. **6**(6), 1955–1968 (2024). 10.1007/s42765-024-00464-y

[10] J. Dong, Y. Peng, Y. Zhang, Y. Chai, J. Long et al., Superelastic radiative cooling metafabric for comfortable epidermal electrophysiological monitoring. Nano-Micro Lett. **15**(1), 181 (2023). 10.1007/s40820-023-01156-9 10.1007/s40820-023-01156-9 PMC10344855 37439918

[11] H. Huang, X. Chu, Y. Xie, B. Zhang, Z. Wang et al., Ti_3_C_2_T_*x*_ MXene-based micro-supercapacitors with ultrahigh volumetric energy density for all-in-one Si-electronics. ACS Nano **16**(3), 3776–3784 (2022). 10.1021/acsnano.1c08172 35239314 10.1021/acsnano.1c08172

[12] Y. Zhang, L. Wang, L. Zhao, K. Wang, Y. Zheng et al., Flexible self-powered integrated sensing system with 3D periodic ordered black Phosphorus@MXene thin-films. Adv. Mater. **33**(22), e2007890 (2021). 10.1002/adma.202007890 33899274 10.1002/adma.202007890

[13] J. Ye, H. Tan, S. Wu, K. Ni, F. Pan et al., Direct laser writing of graphene made from chemical vapor deposition for flexible, integratable micro-supercapacitors with ultrahigh power output. Adv. Mater. **30**(27), e1801384 (2018). 10.1002/adma.201801384 29774618 10.1002/adma.201801384

[14] D. Lu, Y. Chen, Z. Lu, L. Ma, Q. Tao et al., Monolithic three-dimensional tier-by-tier integration *via* van der Waals lamination. Nature **630**(8016), 340–345 (2024). 10.1038/s41586-024-07406-z 38778106 10.1038/s41586-024-07406-z

[15] J.-H. Kang, H. Shin, K.S. Kim, M.-K. Song, D. Lee et al., Monolithic 3D integration of 2D materials-based electronics towards ultimate edge computing solutions. Nat. Mater. **22**(12), 1470–1477 (2023). 10.1038/s41563-023-01704-z 38012388 10.1038/s41563-023-01704-z

[16] S. Pyo, J. Lee, K. Bae, S. Sim, J. Kim, Recent progress in flexible tactile sensors for human-interactive systems: from sensors to advanced applications. Adv. Mater. **33**(47), 2005902 (2021). 10.1002/adma.202005902 10.1002/adma.202005902 33887803

[17] G. Sun, Z. Sun, P. Wang, Z. Zhang, C. Meng et al., Breathable, hydrophobic and antibacterial bioinspired fabric pressure sensors for comfortable skin-mountable health monitoring. Chem. Eng. J. **506**, 159808 (2025). 10.1016/j.cej.2025.159808

[18] J. Dong, Y. Peng, J. Long, Y. Zhang, Z. Wang et al., An all-stretchable, ultraviolet protective, and electromagnetic-interference-free e-textile. Adv. Funct. Mater. **33**(45), 2308426 (2023). 10.1002/adfm.202308426

[19] J. Dong, D. Wang, Y. Peng, C. Zhang, F. Lai et al., Ultra-stretchable and superhydrophobic textile-based bioelectrodes for robust self-cleaning and personal health monitoring. Nano Energy **97**, 107160 (2022). 10.1016/j.nanoen.2022.107160

[20] Y. Liu, J. Liu, S. Chen, T. Lei, Y. Kim et al., Soft and elastic hydrogel-based microelectronics for localized low-voltage neuromodulation. Nat. Biomed. Eng. **3**(1), 58–68 (2019). 10.1038/s41551-018-0335-6 30932073 10.1038/s41551-018-0335-6

[21] F. Bu, W. Zhou, Y. Xu, Y. Du, C. Guan et al., Recent developments of advanced micro-supercapacitors: design, fabrication and applications. NPJ Flex. Electron. **4**, 31 (2020). 10.1038/s41528-020-00093-6

[22] C. Gao, J. Huang, Y. Xiao, G. Zhang, C. Dai et al., A seamlessly integrated device of micro-supercapacitor and wireless charging with ultrahigh energy density and capacitance. Nat. Commun. **12**(1), 2647 (2021). 10.1038/s41467-021-22912-8 33976170 10.1038/s41467-021-22912-8 PMC8113435

[23] H.C. Ates, P.Q. Nguyen, L. Gonzalez-Macia, E. Morales-Narváez, F. Güder et al., End-to-end design of wearable sensors. Nat. Rev. Mater. **7**(11), 887–907 (2022). 10.1038/s41578-022-00460-x 35910814 10.1038/s41578-022-00460-x PMC9306444

[24] H. Liu, C. Du, L. Liao, H. Zhang, H. Zhou et al., Approaching intrinsic dynamics of MXenes hybrid hydrogel for 3D printed multimodal intelligent devices with ultrahigh superelasticity and temperature sensitivity. Nat. Commun. **13**, 3420 (2022). 10.1038/s41467-022-31051-7 35701412 10.1038/s41467-022-31051-7 PMC9197829

[25] J. Dong, J. Hou, Y. Peng, Y. Zhang, H. Liu et al., Breathable and stretchable epidermal electronics for health management: recent advances and challenges. Adv. Mater. **36**(49), 2409071 (2024). 10.1002/adma.202409071 10.1002/adma.202409071 39420650

[26] Y. Peng, J. Dong, J. Long, Y. Zhang, X. Tang et al., Thermally conductive and UV-EMI shielding electronic textiles for unrestricted and multifaceted health monitoring. Nano-Micro Lett. **16**(1), 199 (2024). 10.1007/s40820-024-01429-x 10.1007/s40820-024-01429-x PMC11109083 38771428

[27] W. He, L. Xu, G. Yu, K. Wang, D. Bao et al., Linear enhanced 3D nanofluid force-electric conversion device. Adv. Mater. **37**(8), e2417498 (2025). 10.1002/adma.202417498 39760253 10.1002/adma.202417498

[28] K. Niu, J. Shi, L. Zhang, Y. Yue, M. Wang et al., A self-healing aqueous ammonium-ion micro batteries based on PVA-NH_4_Cl hydrogel electrolyte and MXene-integrated perylene anode. Nano Res. Energy **3**(4), e9120127 (2024). 10.26599/nre.2024.9120127

[29] E. Kim, S. Kim, H.M. Jin, G. Kim, H.-H. Ha et al., Unlocking novel functionality: pseudocapacitive sensing in MXene-based flexible supercapacitors. Nano-Micro Lett. **17**(1), 86 (2024). 10.1007/s40820-024-01567-2 10.1007/s40820-024-01567-2 PMC11628472 39652269

[30] Y. Sun, W. He, C. Jiang, J. Li, J. Liu et al., Wearable biodevices based on two-dimensional materials: from flexible sensors to smart integrated systems. Nano-Micro Lett. **17**(1), 109 (2025). 10.1007/s40820-024-01597-w 10.1007/s40820-024-01597-w PMC11735798 39812886

[31] Y. Xiao, H. Li, T. Gu, X. Jia, S. Sun et al., Ti_3_C_2_T_x_ composite aerogels enable pressure sensors for dialect speech recognition assisted by deep learning. Nano-Micro Lett. **17**(1), 101 (2024). 10.1007/s40820-024-01605-z 10.1007/s40820-024-01605-z PMC11683042 39738742

[32] T. Xu, Q. Song, K. Liu, H. Liu, J. Pan et al., Nanocellulose-assisted construction of multifunctional MXene-based aerogels with engineering biomimetic texture for pressure sensor and compressible electrode. Nano-Micro Lett. **15**(1), 98 (2023). 10.1007/s40820-023-01073-x 10.1007/s40820-023-01073-x PMC10086089 37038023

[33] Z. Yang, S. Lv, Y. Zhang, J. Wang, L. Jiang et al., Self-assembly 3D porous crumpled MXene spheres as efficient gas and pressure sensing material for transient all-MXene sensors. Nano-Micro Lett. **14**(1), 56 (2022). 10.1007/s40820-022-00796-7 10.1007/s40820-022-00796-7 PMC8816976 35122157

[34] M. Wang, Y. Cheng, H. Zhang, F. Cheng, Y. Wang et al., Nature-inspired interconnected macro/meso/micro-porous MXene electrode. Adv. Funct. Mater. **33**(12), 2211199 (2023). 10.1002/adfm.202211199

[35] Y. Cheng, Y. Xie, Y. Ma, M. Wang, Y. Zhang et al., Optimization of ion/electron channels enabled by multiscale MXene aerogel for integrated self-healable flexible energy storage and electronic skin system. Nano Energy **107**, 108131 (2023). 10.1016/j.nanoen.2022.108131

[36] Y. Zhang, Y. Cheng, Q. Zhang, W. He, Y. Wang et al., Hyperstable low-tortuosity fast ion nanochannels for MXene electrodes. Energy Storage Mater. **73**, 103829 (2024). 10.1016/j.ensm.2024.103829

[37] Y. Cheng, Y. Xie, S. Yan, Z. Liu, Y. Ma et al., Maximizing the ion accessibility and high mechanical strength in nanoscale ion channel MXene electrodes for high-capacity zinc-ion energy storage. Sci. Bull. **67**(21), 2216–2224 (2022). 10.1016/j.scib.2022.10.003 10.1016/j.scib.2022.10.003 36545997

[38] S. Zheng, Q. Tu, J.J. Urban, S. Li, B. Mi, Swelling of graphene oxide membranes in aqueous solution: characterization of interlayer spacing and insight into water transport mechanisms. ACS Nano **11**(6), 6440–6450 (2017). 10.1021/acsnano.7b02999 28570812 10.1021/acsnano.7b02999

[39] Z. Wu, Y. Deng, J. Yu, J. Han, T. Shang et al., Hydroiodic-acid-initiated dense yet porous Ti_3_C_2_T_*x*_ MXene monoliths toward superhigh areal energy storage. Adv. Mater. **35**(29), 2300580 (2023). 10.1002/adma.202300580 10.1002/adma.202300580 37037650

[40] W. Zhang, Y. Lu, L. Wan, P. Zhou, Y. Xia et al., Engineering a passivating electric double layer for high performance lithium metal batteries. Nat. Commun. **13**(1), 2029 (2022). 10.1038/s41467-022-29761-z 35440573 10.1038/s41467-022-29761-z PMC9018679

[41] Y. Luo, Q. Zhang, W. Hong, Z. Xiao, H. Bai, A high-performance electrochemical supercapacitor based on a polyaniline/reduced graphene oxide electrode and a copper(ii) ion active electrolyte. Phys. Chem. Chem. Phys. **20**(1), 131–136 (2018). 10.1039/C7CP07156F 10.1039/c7cp07156f 29210393

[42] H.A. Andreas, K. Lussier, A.M. Oickle, Effect of Fe-contamination on rate of self-discharge in carbon-based aqueous electrochemical capacitors. J. Power. Sources **187**(1), 275–283 (2009). 10.1016/j.jpowsour.2008.10.096

[43] Q. Zhang, C. Cai, J. Qin, B. Wei, Tunable self-discharge process of carbon nanotube based supercapacitors. Nano Energy **4**, 14–22 (2014). 10.1016/j.nanoen.2013.12.005

[44] M. Xia, J. Nie, Z. Zhang, X. Lu, Z.L. Wang, Suppressing self-discharge of supercapacitors *via* electrorheological effect of liquid crystals. Nano Energy **47**, 43–50 (2018). 10.1016/j.nanoen.2018.02.022

[45] J. Wang, B. Ding, X. Hao, Y. Xu, Y. Wang et al., A modified molten-salt method to prepare graphene electrode with high capacitance and low self-discharge rate. Carbon **102**, 255–261 (2016). 10.1016/j.carbon.2016.02.047

[46] F.O. Ochai-Ejeh, M.J. Madito, D.Y. Momodu, A.A. Khaleed, O. Olaniyan et al., High performance hybrid supercapacitor device based on cobalt manganese layered double hydroxide and activated carbon derived from cork (*Quercus suber*). Electrochim. Acta **252**, 41–54 (2017). 10.1016/j.electacta.2017.08.163

[47] Y. Yue, Z. Yang, N. Liu, W. Liu, H. Zhang et al., A flexible integrated system containing a microsupercapacitor, a photodetector, and a wireless charging coil. ACS Nano **10**(12), 11249–11257 (2016). 10.1021/acsnano.6b06326 28024378 10.1021/acsnano.6b06326

[48] Q. Han, X. Chi, S. Zhang, Y. Liu, B. Zhou et al., Durable, flexible self-standing hydrogel electrolytes enabling high-safety rechargeable solid-state zinc metal batteries. J. Mater. Chem. A **6**(45), 23046–23054 (2018). 10.1039/c8ta08314b

[49] J. Liu, B. Zhang, P. Zhang, K. Zhao, Z. Lu et al., Protein crystallization-mediated self-strengthening of high-performance printable conducting organohydrogels. ACS Nano **16**(11), 17998–18008 (2022). 10.1021/acsnano.2c07823 36136126 10.1021/acsnano.2c07823

[50] C. Wang, Z. Chen, C.L.J. Chan, Z. Wan, W. Ye et al., Biomimetic olfactory chips based on large-scale monolithically integrated nanotube sensor arrays. Nat. Electron. **7**(2), 157–167 (2024). 10.1038/s41928-023-01107-7

[51] A. Tashakori, Z. Jiang, A. Servati, S. Soltanian, H. Narayana et al., Capturing complex hand movements and object interactions using machine learning-powered stretchable smart textile gloves. Nat. Mach. Intell. **6**(1), 106–118 (2024). 10.1038/s42256-023-00780-9

