# Supplementary material for: Flexible Monolithic 3D-Integrated Self-Powered Tactile Sensing Array Based on Holey MXene Paste
Source: Nanomicro Lett. 2025 Sep 15;18:68. doi: 10.1007/s40820-025-01924-9 (PMC12436258; doi:10.1007/s40820-025-01924-9)
Supplement: Supplementary file 3 — Supplementary file3 (DOCX 12924 KB) [file 40820_2025_1924_MOESM3_ESM.docx]

Supporting Information for

**Flexible Monolithic 3D Integrated Self-Powered Tactile Sensing Array Based on Holey MXene Paste**

Mengjie Wang^1†^, Chen Chen^1†^, Yuhang Zhang^1†^, Yanan Ma^2*^, Li Xu^2*^, Dan-Dan Wu^1^, Bowen Gao^2^, Aoyun Song^1^, Li Wen^1^, Yongfa Cheng^3^, Siliang Wang^1*^, Yang Yue^1*^

^1^ Information Materials and Intelligent Sensing Laboratory of Anhui Province, Industry-Education-Research Institute of Advanced Materials and Technology for Integrated Circuits, Institutes of Physical Science and Information Technology, Anhui University, Hefei 230601, People’s Republic of China

^2^ Hubei key laboratory of energy storage and power battery, School of Optoelectronic Engineering, Hubei University of Automotive Technology, Shiyan 442002, People’s Republic of China

^3^ Department of Materials Science and Engineering, Northwestern University, 2145 Sheridan Road, Evanston, IL 60208, USA

† Mengjie Wang, Chen Chen, and Yuhang Zhang contributed equally to this work.

* Corresponding author. E-mail: mayn@huat.edu.cn (Yanan Ma); [xuli@huat.edu.cn](mailto:xuli@huat.edu.cn) (Li Xu); [sliang_wang@163.com](mailto:sliang_wang@163.com) (Siliang Wang); [yueyang@ahu.edu.cn](mailto:yueyang@ahu.edu.cn) (Yang Yue)

**S1 Calculation of electrostatic repulsive and van der Waals potential energy based on the electric double layer theory**

Based on the formation of an electrical double layer, the Debye length can be calculated by:

$$\lambda_{D}=\sqrt{\frac{\varepsilon_{r}\varepsilon_{0}kT}{2N_{A}e^{2}I}}$$

$$I=\frac{1}{2}(n_{+}z_{+}^{2}+n_{-}z_{-}^{2})C_{0}$$

where *ε_r_* is the relative permittivity of water (80.1 at room temperature), *ε_0_* is the vacuum permittivity (8.85×10^-12^ C^2^/J·m), *k* is Boltzmann constant (1.38×10^-23^ J/K), *T* is the absolute temperature (298 K), *N_A_* is Avogadro number (6.02×10^23^), *e* is the electron charge (1.6×10^-19^), and *I* is the ionic strength of the bulk solution, *n_+_* and *n_-_* are the particle numbers of positive and negative ions generated by the electrolyte; *z_+_* and *z_-_* are the valence numbers of positive and negative ions, and *C_0_* is the electrolyte concentration of the bulk solution.

The van der Waals potential energy *E_A_* is calculated as follows:

$$E_{A}=-\frac{A}{12\pi}\left[ \frac{1}{D^{2}}-\frac{2}{\left( D+d \right)^{2}}+\frac{1}{\left( D+2d \right)^{2}} \right]$$

where *d* are the thicknesses of the nanosheets and *D* is the nanosheet interlayer distance; *A* is the Hamaker constant, is calculated as the following calculation.

$$A=\frac{3kT}{4}{(\frac{\varepsilon-\varepsilon_{HCl}}{\varepsilon+\varepsilon_{HCl}})}^{2}+\frac{3h\omega}{16\sqrt{2}}\frac{{(n^{2}-n_{HCl}^{2})}^{2}}{{(n^{2}+n_{HCl}^{2})}^{3/2}}$$

where *k* is the Boltzmann constant when *T* = 298 K, *ε* is the dielectric constant of holey MXene, *n* is the refractive index of holey MXene. *ε_HCl_* is the dielectric constant of HCl, *n_HCl_* is the refractive index of HCl, *h* is the Planck’s constant, and *ω* = 4.73×10^14^ s^−1^ the absorption frequency.

The electrostatic repulsive potential energy *E_R_* is calculated as follows:

$$E_{R}=\frac{64N_{A}IkT}{\kappa}\left\{ tanh\left( \frac{{e\psi}_{0}}{4kT} \right) \right\}^{2}exp\left( -\kappa D \right)$$

$$\kappa^{-1}=\lambda_{D}$$

*ψ_0_* is the surface potential and *κ* is the Debye Huckel parameter.

Therefore, the total potential energy *E_total_* can be calculated:

$$E_{total}=E_{A}+E_{R}$$

**S2 Supplementary Figures and Tables**


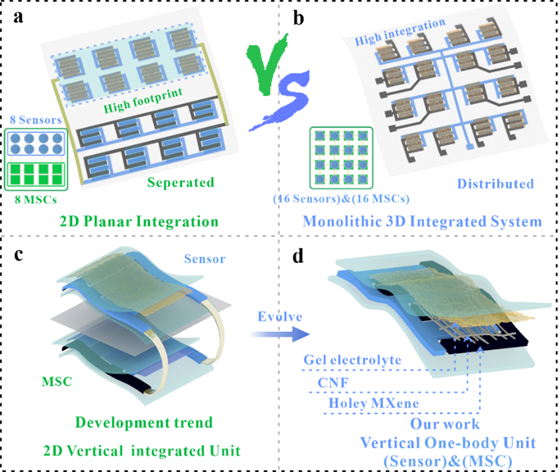


**Fig. S1** The development of the all-in-one integration and the advantages of 3D vertically integrated systems

Compared to traditional 2D planar integration (Fig. S1a), our overall 3D monolithic integration strategy offers significant advantages in terms of area efficiency and vertical compactness (Fig. S1b), which can avoid the inherent routing challenge and additional space requirements associated with 2D interconnections. Specific advantages are as follows: **i) Area Efficiency:** Within the same footprint, the VOU can accommodate at least twice the number of devices, effectively reducing the area required for equivalent functionality by more than 50%. **ii) Vertical Compactness:** As shown in Figs. S1c, d, traditional 2D vertical stacking requires separate current collectors and encapsulation layers for each device layer. In contrast, our strategy uses shared current collectors and common encapsulation layers, reducing the total stack height by at least 50% and doubling the volume density. **iii) Simplified interconnections:** The inherent shared current collector in VOU eliminates the need for discrete wiring between integrated components (sensors and capacitors), significantly shortening signal paths and overcoming the interconnection challenges common in traditional 2D designs (Figs. S1c, d). The reduced signal paths contribute to lower power consumption and faster response times. The improved spatial efficiency and device density significantly enhance the sensing performance and integration capability of the VOU system.


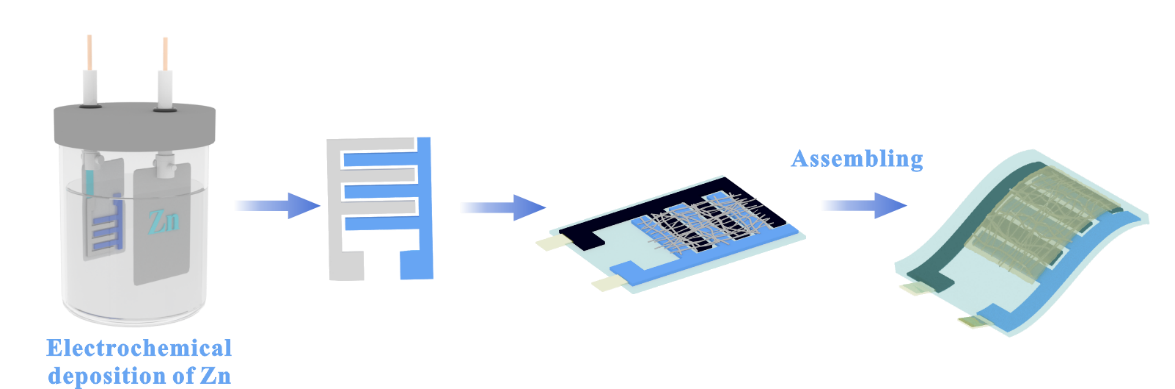
**Fig. S2** The flow diagram for assembling the VOU

**
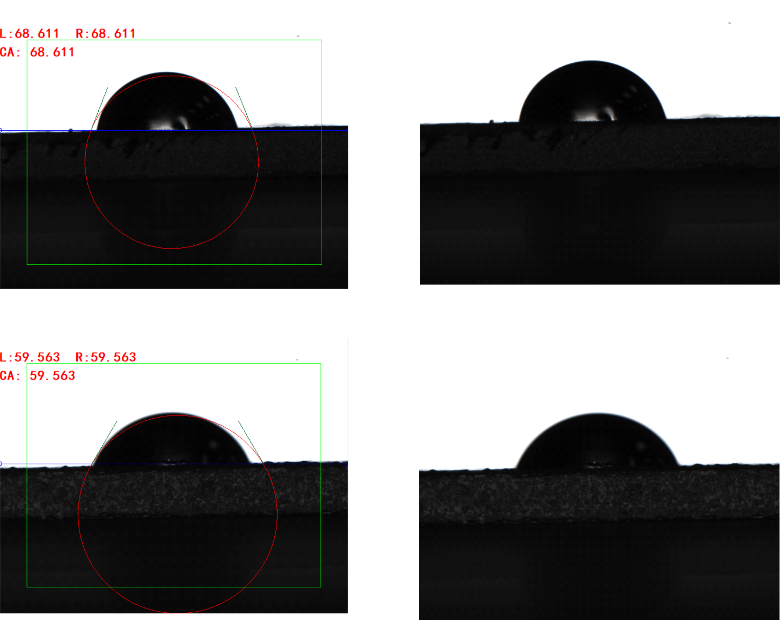
**

**Fig. S3** Contact angle tests of MXene and holey MXene materials


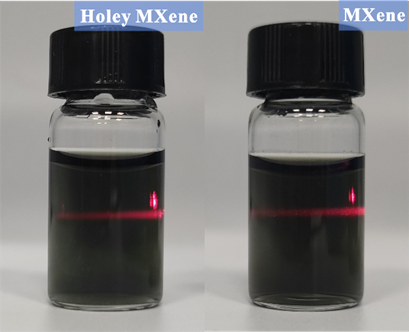


**Fig. S4** Tyndall effect of MXene and holey MXene solution

**
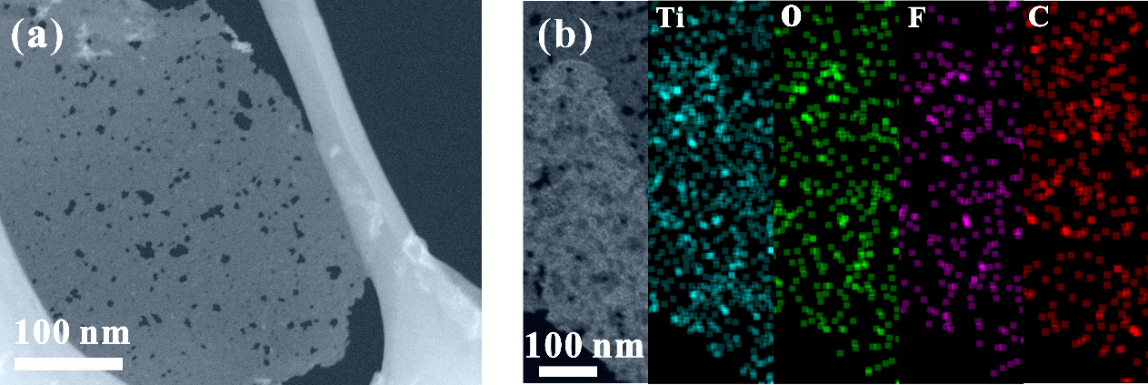
**

**Fig. S5** HAADF-STEM images, and EDS mapping of the holey MXene nanosheets

The HAADF-STEM images clearly show the holes fabricated by etching. And the vacancy defects are also formed during the etching process, which provides a large number of active sites and shorter ionic transport distance, thus improving the electrochemical performance. And the corresponding EDS show a uniform distribution of Ti, O, C and F elements in the holey MXene nanosheets.


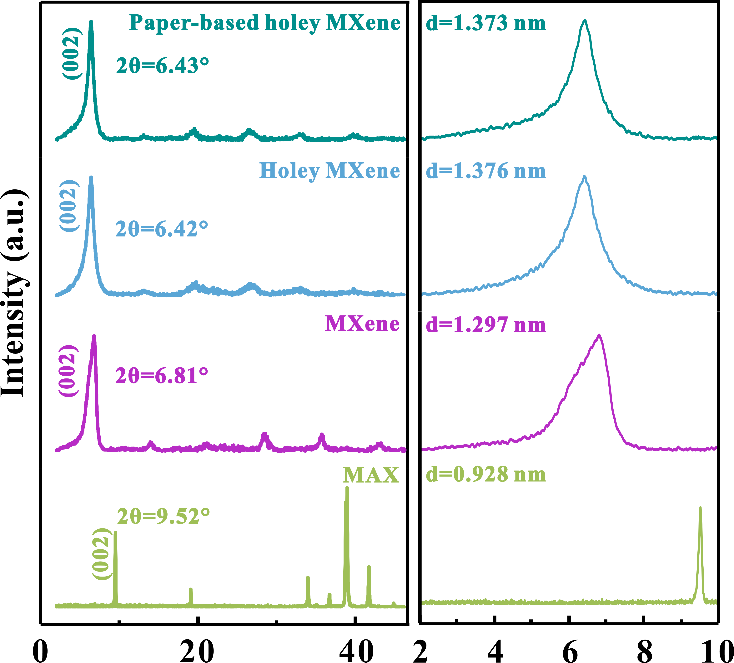


**Fig. S6** XRD spectra of MAX powder, MXene film, holey MXene film, and holey MXene based paper

For XRD characterization analysis, the (002) diffraction peak of the MAX phase was shifted from 9.52° to 6.81° after chemical etching, and the (104) diffraction peak at 39° disappeared, proving the successful preparation of MXene. And the interlayer distance can be calculated based on the Bragg's equation (2dsinθ = nλ). The interlayer spacing for MAX phase, pristine MXene, holey MXene, and holey MXene based paper were 0.928 nm, 1.297 nm, 1.376 nm, and 1.373 nm, respectively. The results show that the introduction of mesopores further enlarges the layer spacing of the nanosheets, effectively reducing the self-stacking phenomenon of MXene nanosheets. It is observed that the addition of trace amounts of HCl does not significantly affect the distance of MXene.


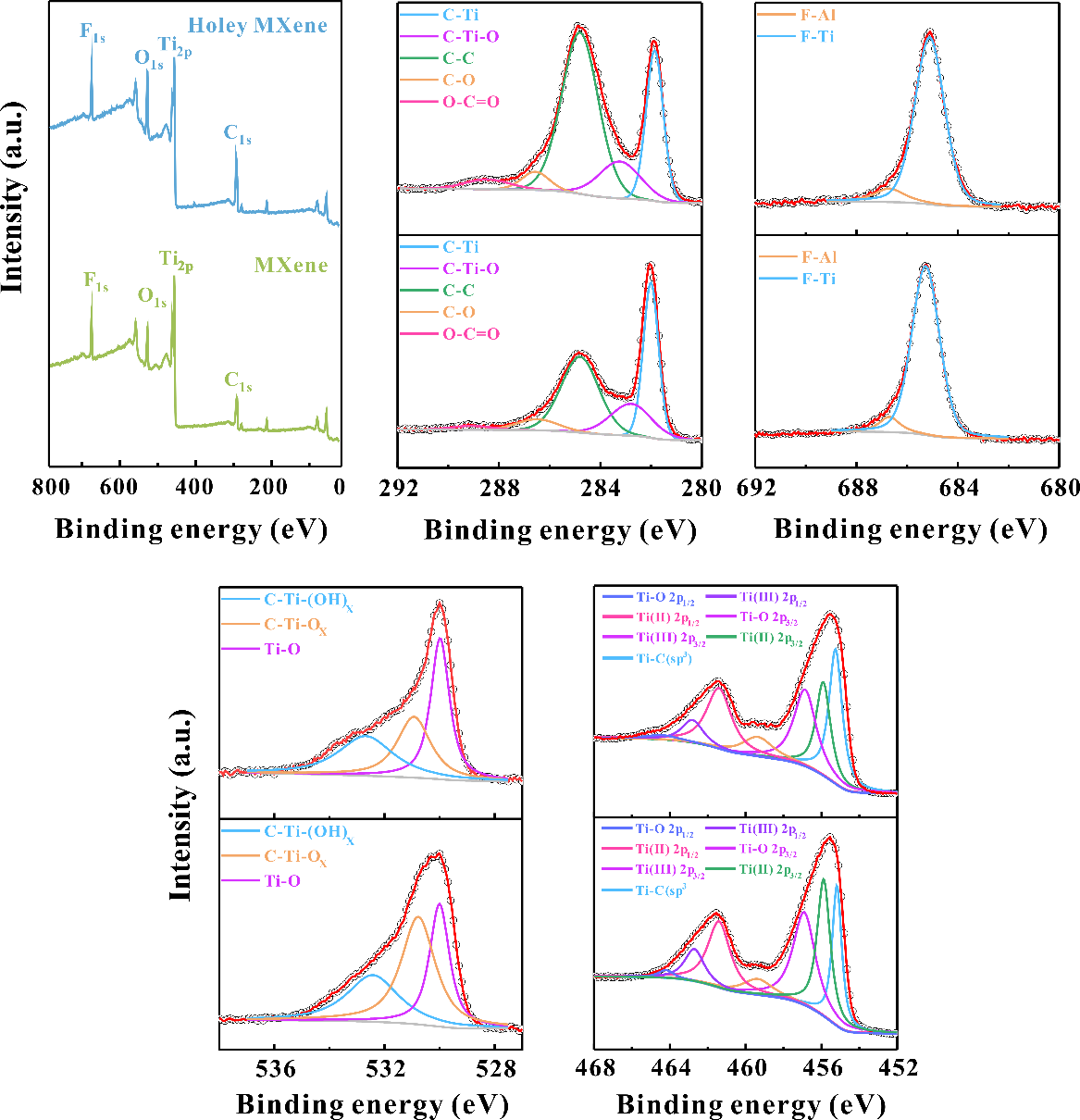


**Fig. S7** XPS spectra of the MXene and holey MXene, and the corresponding high-resolution spectra of C 1s, F 1s, O 1s, and Ti 2p

The XPS spectra of pristine and holey MXene detected F 1s, O 1s, Ti 2p, and C 1s elements. In C1s spectra, the intensity of C-C peaks of the holey MXene is significantly higher, indicating that more C atoms are exposed due to the introduction of mesopores. In addition, both C-Ti bonds remained after *in-situ* etching, indicating the presence of the layered structure. And all Ti 2p spectra showed weak Ti-O peaks, indicating the effective removement of TiO_2_.


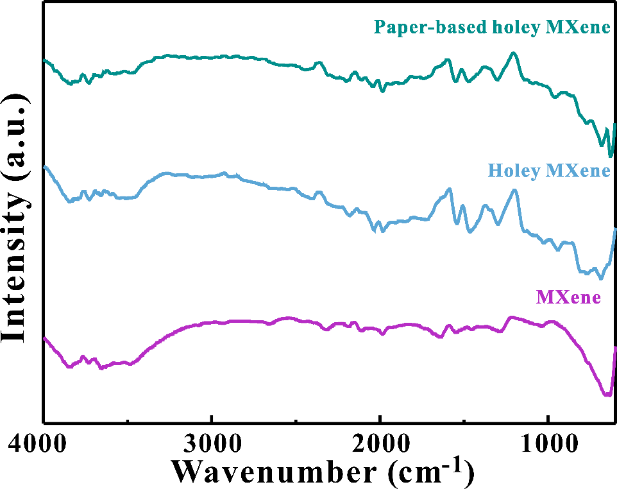


**Fig.** **S8** FTIR spectra of MXene, holey MXene, and holey MXene based paper

Both the MXene and holey MXene mainly shows three characteristic peaks of -OH, -C=O, and -Ti-O, which is consistent with the reported work.

**
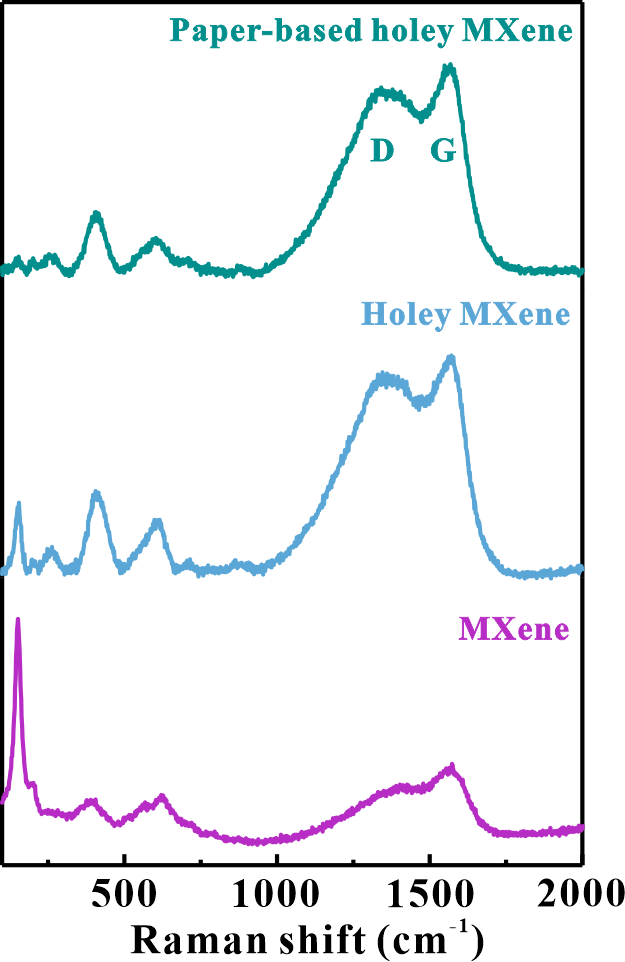
**

**Fig.** **S9** Raman spectra of MXene, holey MXene, and holey MXene based paper

The results show the distinct D and G bands after H_2_O_2_ etching, attributed to the formation of carbon during etching the holes.

**
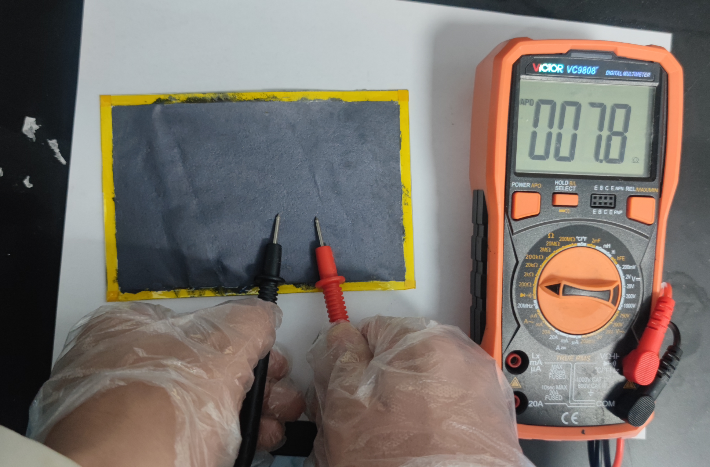
**

**Fig. S10** The holey MXene based A4 paper overall maintains good electrical conductivity


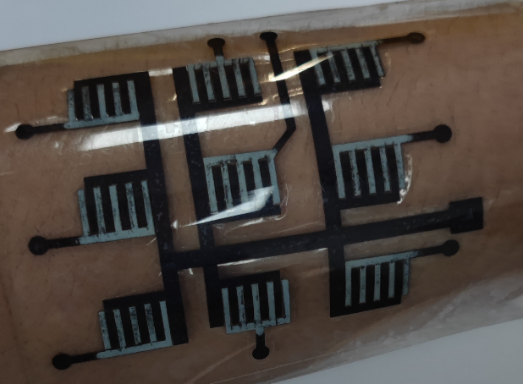


**Fig. S11** The 3*3 monolithic 3D integrated sensing systems attached to the arm, demonstrating good flexibility


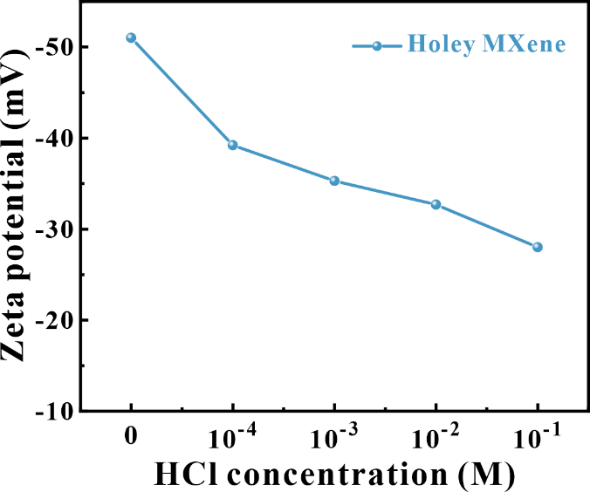


**Fig. S12** Zeta potential of holey MXene without/with H^+^ (2.5 mL of 10^-4^, 10^-3^, 10^-2^, and 10^-1^ M HCl solutions were added to 6 mL 0.3 mg mL^-1^ MXene solution, respectively)


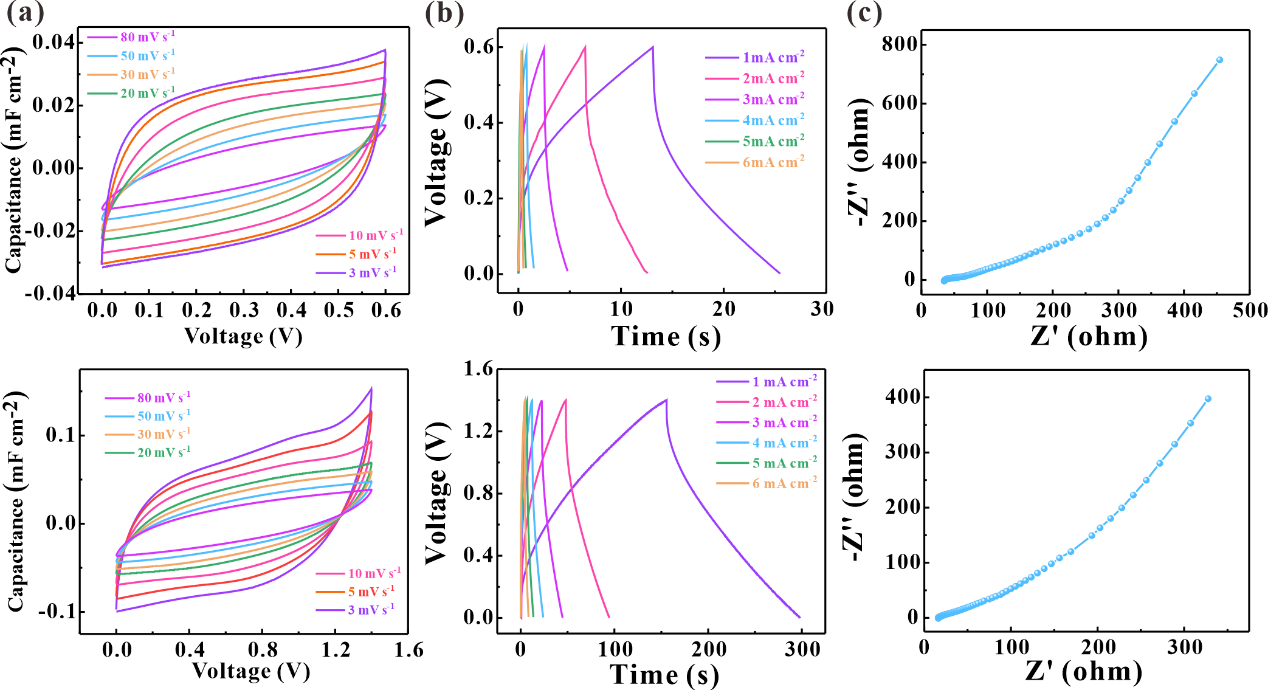


**Fig. S13 a** CV curves of the VOU at 3-80 mV s^-1^, **b** the corresponding GCD curves at 1-6 mA cm^-2^, and **c** EIS curves

**
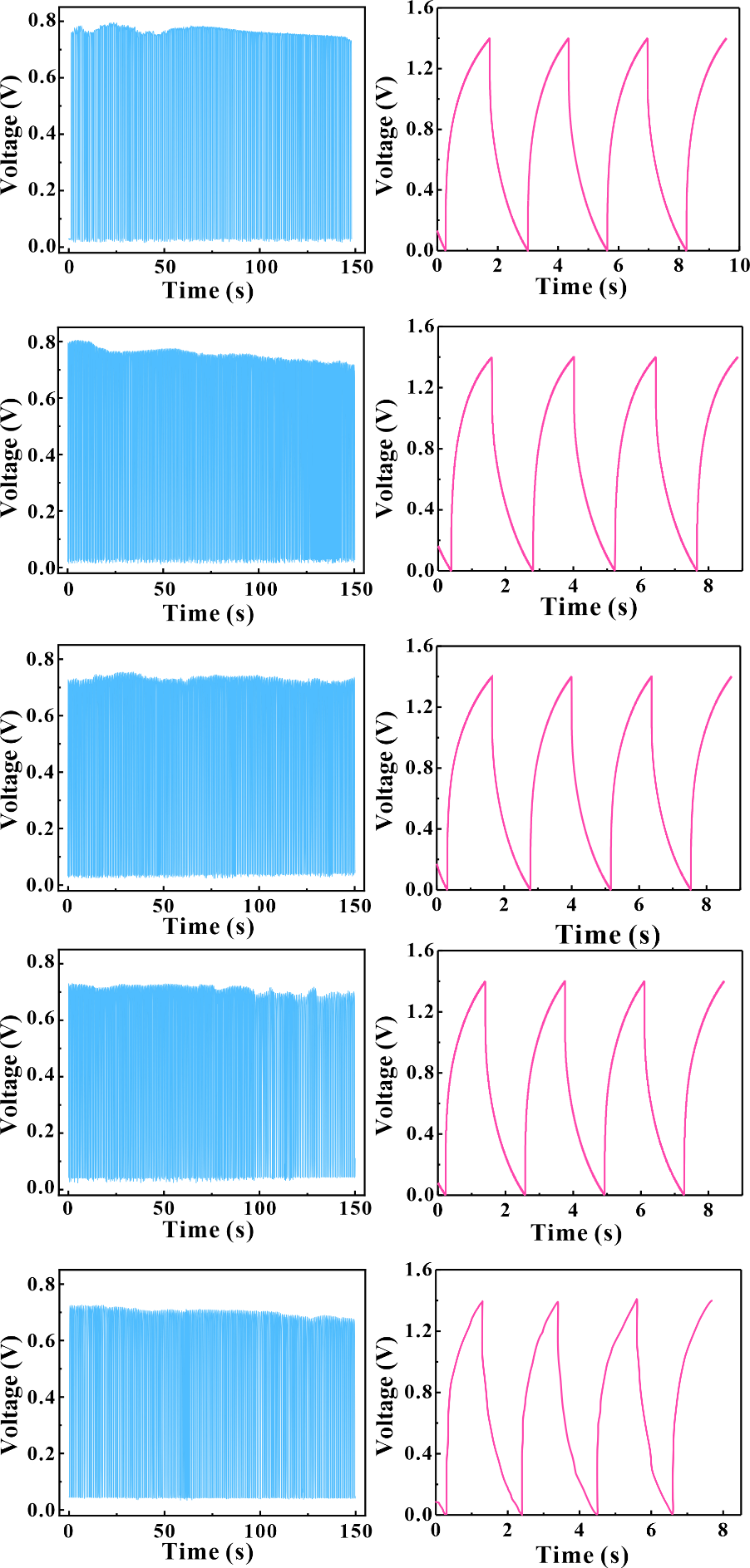
**

**Fig. S14** The charge/discharge tests of the VOU after every 200 cycles, owning the reliable electrochemical performance

**
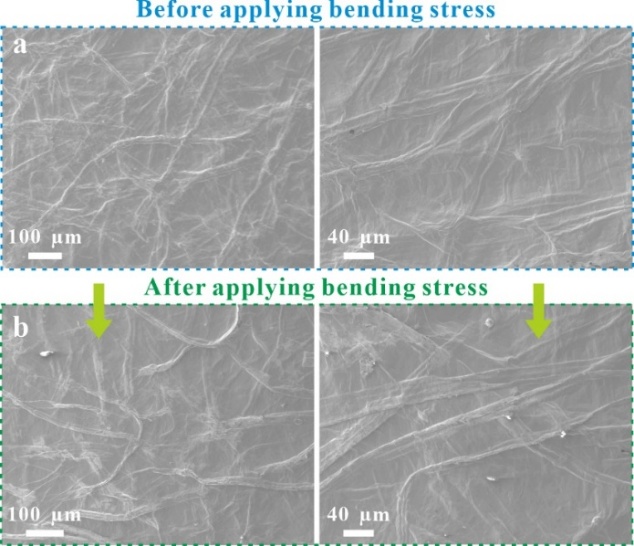
**

**Fig. S15** SEM images of the surface microstructure of the VOU under stress conditions (bending)


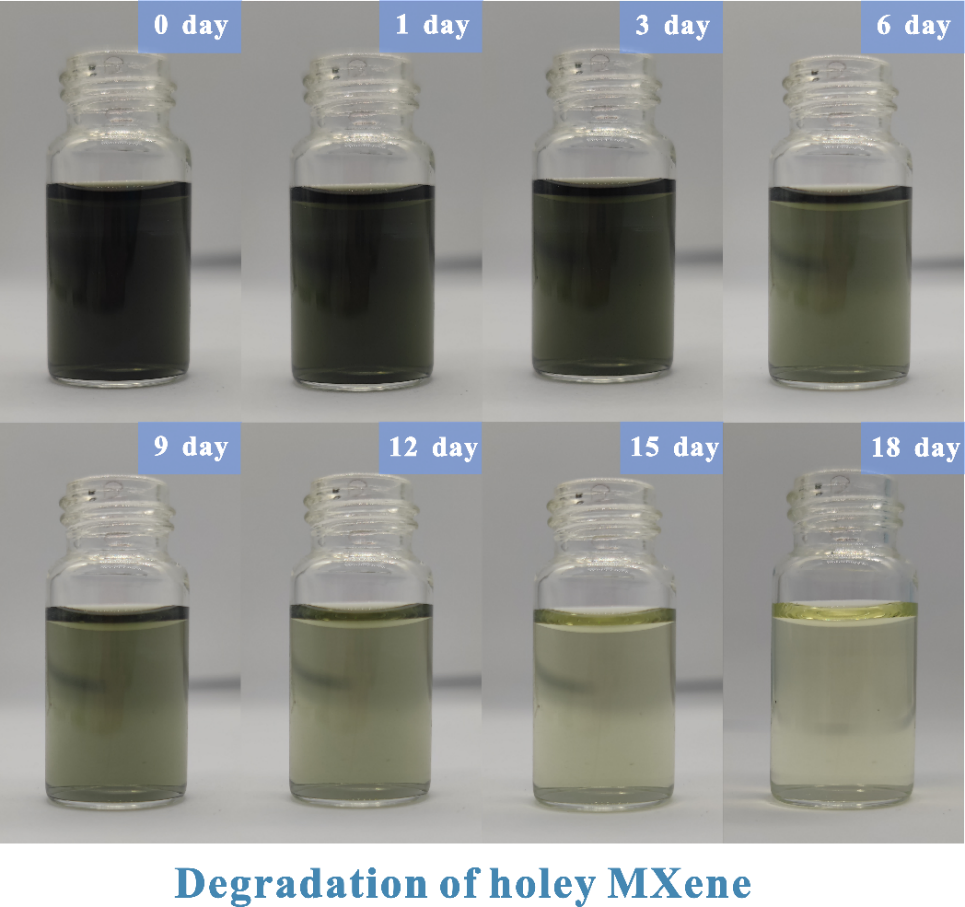


**Fig. S16** Schematic diagram of the self-degradation process of holey MXene solution


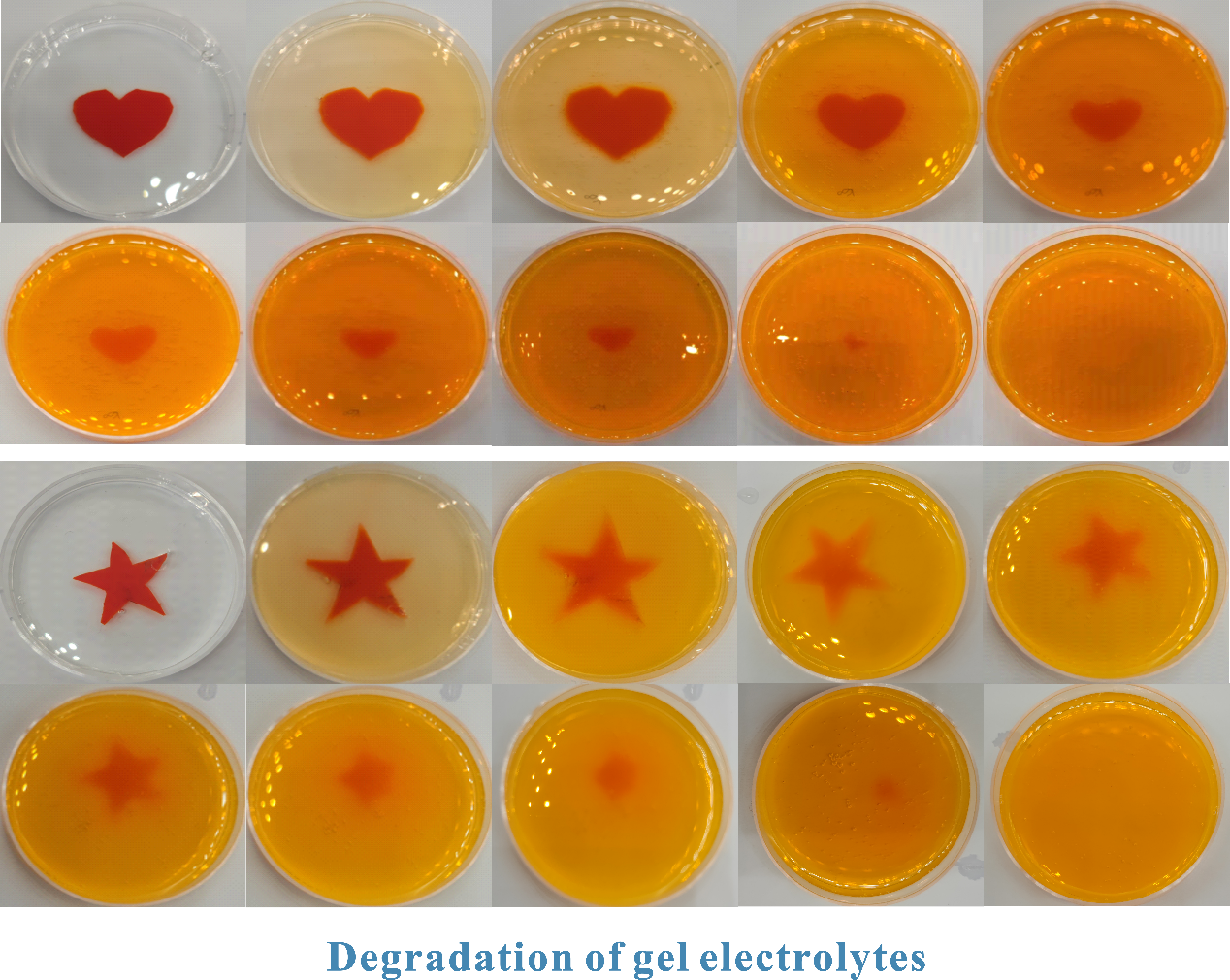
**Fig. S17** Degradation process of the gel electrolyte, colored by red ink for easy observation


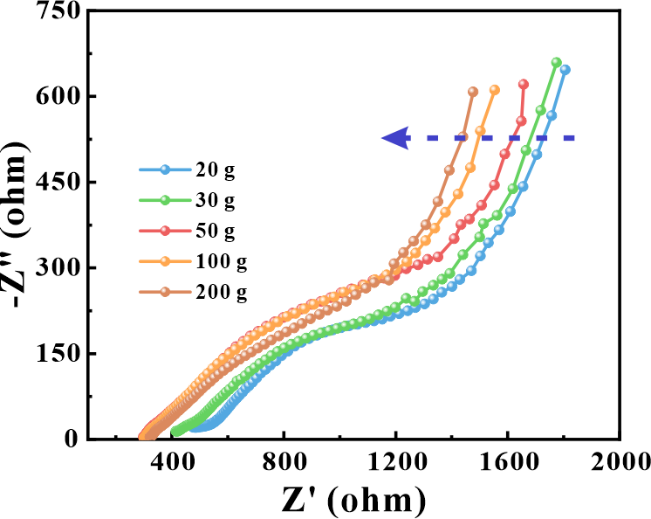


**Fig. S18** EIS spectra of the VOU


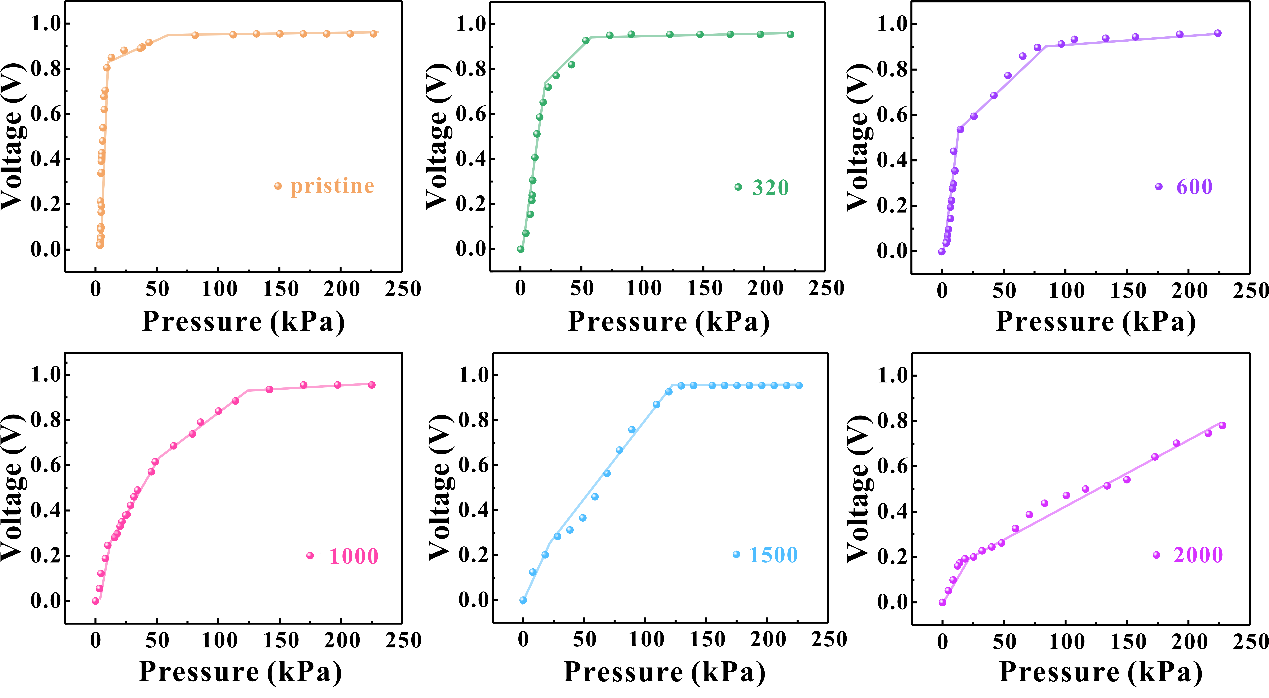


**Fig. S19** The sensing performance of the VOU with gel electrolytes molding 320, 600, 1000, 1500, and 2000 mesh sandpapers, respectively

As shown in Fig. S19, by using sandpapers with mesh numbers ranging from 320 to 2000 mesh, we successfully constructed microstructures with different roughness levels on the surface of the gel electrolyte. This microstructure can synergistically regulate the sensitivity and pressure detection range of VOU. The experimental results indicate that as the mesh number of the sandpaper increases, the sensitivity of the VOU slightly decreases, but the pressure detection range significantly widens. The widening of the pressure detection range is primarily attributed to the rich microstructures in high-mesh sandpapers. The original gel surface lacks microstructures, which causes the contact area to rapidly saturate under pressure, in turn resulting in narrow detection range. As the mesh number of the sandpaper increases, more complex microstructures are introduced into the gel, significantly delaying the saturation point of the contact area and increasing the pressure required to reach saturation. Consequently, both the pressure detection and linear response range of the sensor synchronously improve with increasing mesh number of the sandpaper.

**
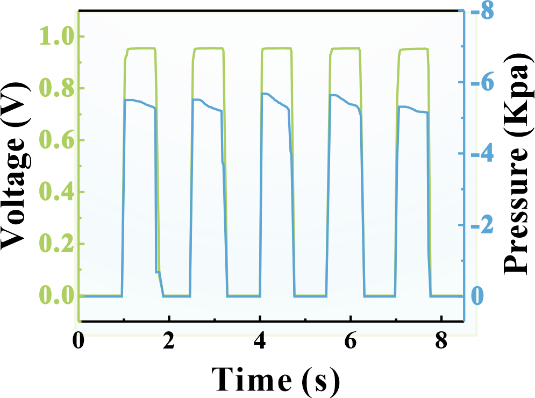
**

**Fig. S20** The response of *V-T* and *P-T* curves of the VOU under periodic loading and unloading conditions


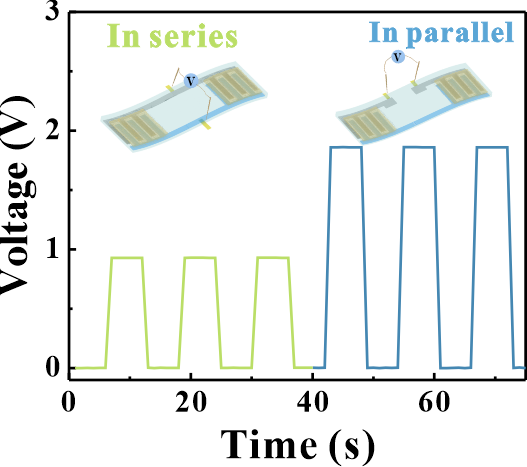


**Fig. S21** The *V-T* curves of the VOUs connected in series and parallel, it conforms to the basic law of series-parallel connection


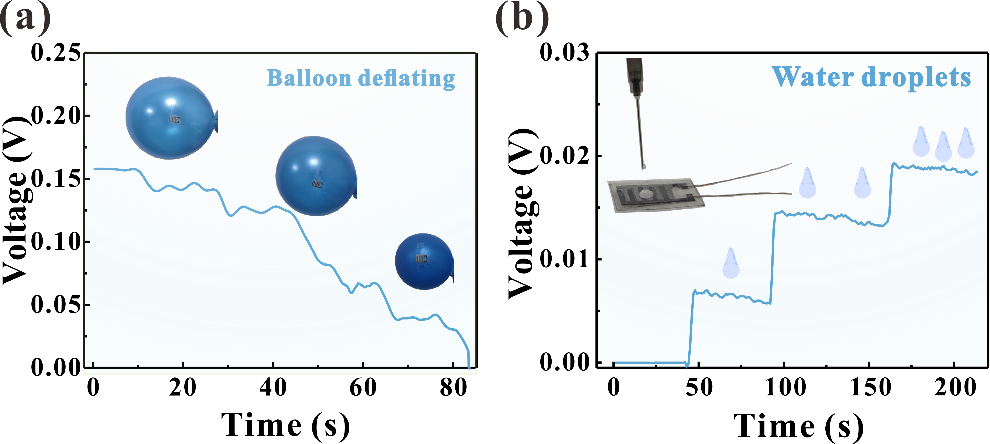


**Fig. S22** The VOU is used to monitor **a** balloon deflation and **b** water droplet, indicating the accurate detection for small pressures


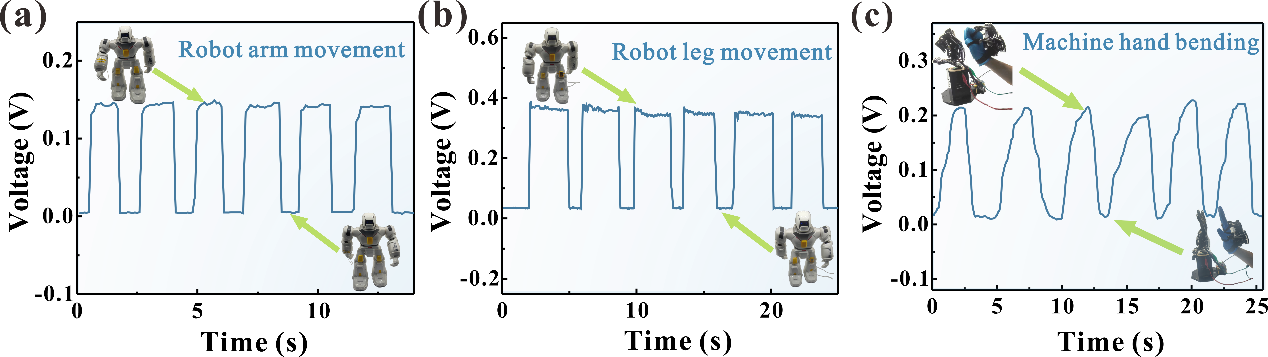
**Fig. S****23** The *V-T* curves of VOU mounted on **a** the arm, **b** the leg of the robot, and **c** the mechanical hand


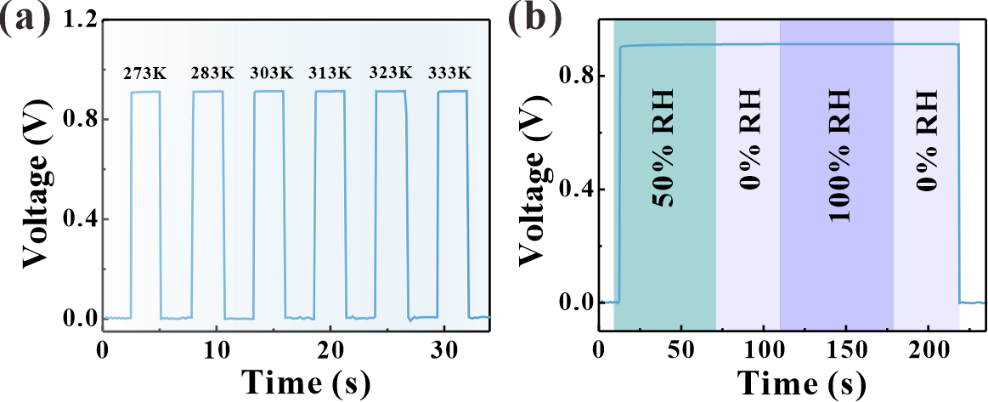


**Fig. S24** **a, b** Force-electric response of the VOU under different temperatures and humidities, showcasing good stability


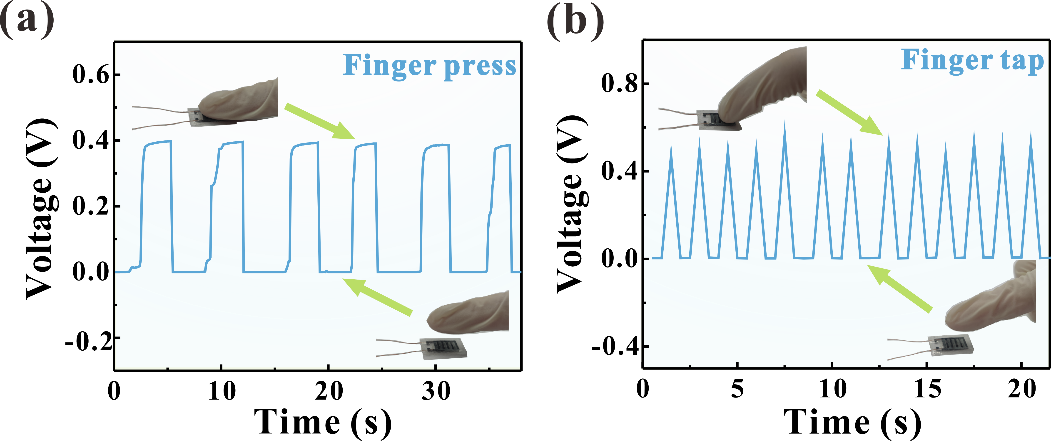


**Fig. S25 a, b** The *V-T* curves of the VOU for finger tapping and finger pressing

After fixing the VOU, it was subjected to finger tapping (high-frequency signals) and finger pressing (continuous signals). Each action response is consistent, showcasing the accurate response.


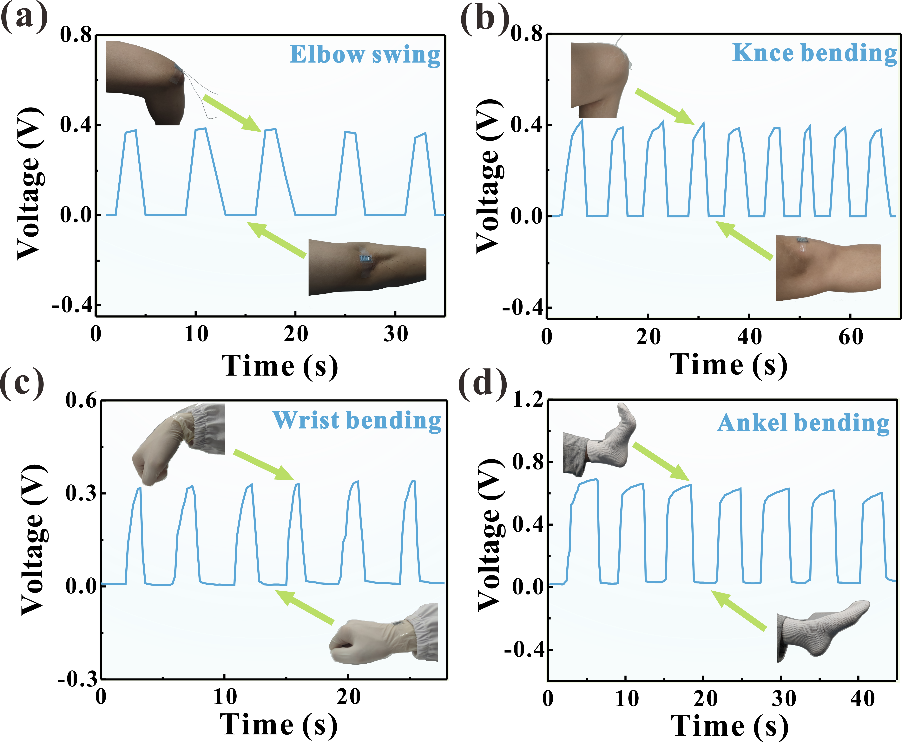


**Fig. S26** The *V-T* curves of the VOU for human motion monitoring **a** elbow swing, **b** knee flexion, **c** wrist pulse, and **d** ankle flexion, respectively

The stable and reliable voltage response demonstrates the significant advantages of movements monitoring of the VOU.


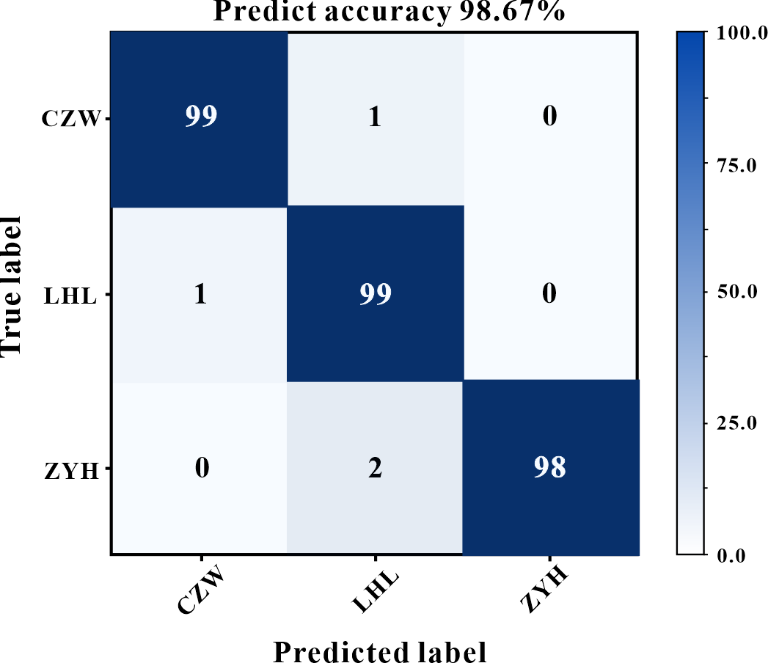


**Fig. S27** Confusion matrix for the accuracy recognition for user tests

**Table S1** The comparisons of VOU with current state-of-the-art sensors

| Materials | Sensitivity | Response Time | Cyclic Stability | Sensor type | References |
| --- | --- | --- | --- | --- | --- |
| MXene/BC | 51.14 mV/kPa | 99 ms | 5000 | Piezoresistive | [S1] |
| rGO/GO/PVA | 5 mV/kPa | 41 ms | 5000 | Potentiometric Transduction | [S2] |
| Graphene/BC | 51.4 mV/kPa | 300 ms | 200 | Piezoresistive | [S3] |
| Ti_3_C_2_T_x_ | 28.43 kPa^-1^ | 98.3 ms | 5000 | Piezoresistive | [S4] |
| ACNT/graphene | 19.8 kPa^-1^ | 16.7 ms | 35000 | Piezoresistive | [S5] |
| VO_2_(B)/PVDF-HFP-GO/Zn | 4.92 mV/kPa | 76 ms | 100000 | Battery type | [S6] |
| MoO_3_/GO[CNF/Ca]/AC | 77.5 mV/kPa | 86 ms | 20000 | Battery type | [S7] |
| Holey MXene | **80 mV/kPa** | **49 ms** | **80000** | **Potentiometric Transduction** | **This work** |

**Supplementary References**

1. T. Su, N. Liu, D. Lei, L. Wang, Z. Ren et al., Flexible MXene/bacterial cellulose film sound detector based on piezoresistive sensing mechanism. ACS Nano **16**(5), 8461–8471 (2022). <https://doi.org/10.1021/acsnano.2c03155>
2. D. Lei, Q. Zhang, N. Liu, T. Su, L. Wang et al., An ion channel-induced self-powered flexible pressure sensor based on potentiometric transduction mechanism. Adv. Funct. Mater. **32**(5), 2108856 (2022). <https://doi.org/10.1002/adfm.202108856>
3. J. Sun, K. Xiu, Z. Wang, N. Hu, L. Zhao et al., Multifunctional wearable humidity and pressure sensors based on biocompatible graphene/bacterial cellulose bioaerogel for wireless monitoring and early warning of sleep apnea syndrome. Nano Energy **108**, 108215 (2023). <https://doi.org/10.1016/j.nanoen.2023.108215>
4. Y. Ma, Y. Cheng, J. Wang, S. Fu, M. Zhou et al., Flexible and highly-sensitive pressure sensor based on controllably oxidized MXene. InfoMat **4**(9), e12328 (2022). <https://doi.org/10.1002/inf2.12328>
5. M. Jian, K. Xia, Q. Wang, Z. Yin, H. Wang et al., Flexible and highly sensitive pressure sensors based on bionic hierarchical structures. Adv. Funct. Mater. **27**(9), 1606066 (2017). <https://doi.org/10.1002/adfm.201606066>
6. Q. Zhang, D. Lei, N. Liu, Z. Liu, Z. Ren et al., A zinc-ion battery-type self-powered pressure sensor with long service life. Adv. Mater. **34**(40), 2205369 (2022). <https://doi.org/10.1002/adma.202205369>
7. Q. Zhang, D. Lei, J. Shi, Z. Ren, J. Yin et al., Pressure-regulated nanoconfined channels for highly effective mechanical–electrical conversion in proton battery-type self-powered pressure sensor. Adv. Mater. **35**(52), 2308795 (2023). <https://doi.org/10.1002/adma.202308795>
